# Recent advances in ferroelectric materials, devices, and in-memory computing applications

**DOI:** 10.1186/s40580-025-00520-2

**Published:** 2025-11-06

**Authors:** Hwiho Hwang, Sangwook Youn, Hyungjin Kim

**Affiliations:** Division of Materials Science and Engineering and Department of Semiconductor Engineering, Hanyang University, Seoul, 04763 Korea

**Keywords:** Ferroelectric thin films, Non-volatile memory devices, In-memory computing, Neuromorphic computing, Hardware security

## Abstract

Ferroelectric memories have undergone a transformative evolution from conventional perovskite-based materials to modern fluorite-structured ferroelectrics, driven by the pursuit of scalable, low-power, and CMOS-compatible non-volatile memory solutions. The observation of ferroelectricity in nanoscale HfO_2_-based films has enabled integration with CMOS-compatible processes, providing advantages such as potential scalability, low power consumption, and non-volatility, while facilitating continued scaling and high-density integration. Leveraging established materials infrastructure in the semiconductor industry, hafnia–based ferroelectrics have been incorporated in various memory architectures, including ferroelectric random-access memory (FeRAM), ferroelectric tunnel junctions (FTJs), ferroelectric field-effect transistors (FeFETs), and ferroelectric memcapacitors (FeCAPs). Beyond conventional non-volatile storage, these devices have also emerged as promising building blocks for in-memory computing applications, including neuromorphic systems, hardware security primitives, and associative memory. In this review, we explore the historical development of ferroelectric memories from a materials–device co-design perspective, examine recent advances in device architectures and in-memory computing applications, and discuss the remaining challenges in endurance, retention, variability, and scaling. Finally, we propose future research directions that integrating material innovation, interface engineering, and circuit-level optimization to realize the full potential of ferroelectric memories in next-generation computing platforms.

**Graphical abstract:**

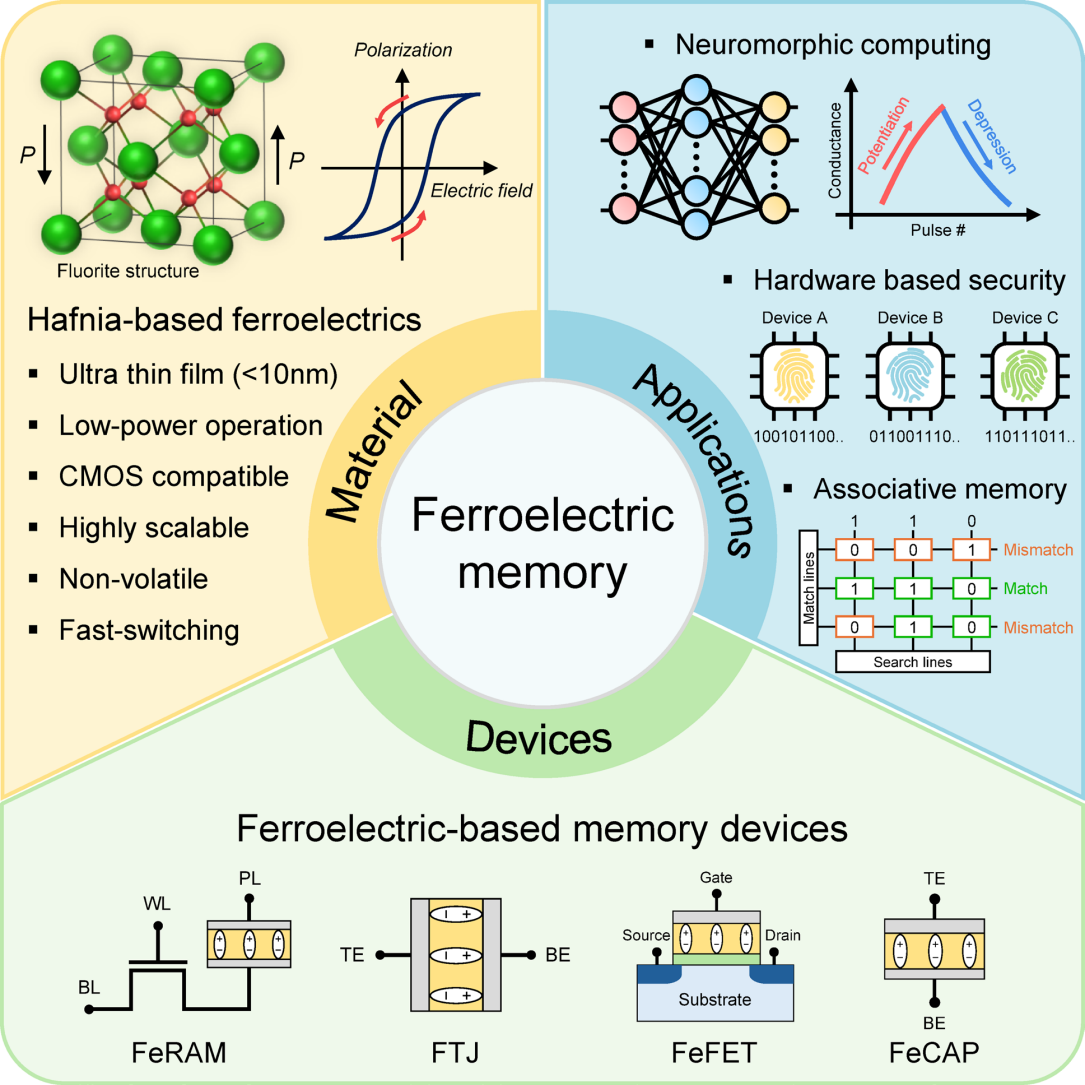

## Introduction

Ferroelectricity is the property of having multiple stable polarization states, arising from a non-centrosymmetric crystal structure. This feature enables spontaneous polarization without an external electric field, and allows reversible switching under an applied field. Owing to these properties, ferroelectric (FE) materials have long been regarded as promising candidates for next-generation nonvolatile memory devices [1–3]. However, the advantageous characteristics of conventional perovskite-based ferroelectrics could not be preserved upon scaling to nanoscale films integrated on silicon, due to polarization degradation, poor CMOS compatibility, and reliability concerns such as fatigue and imprint. These limitations have long hindered the realization of practical ferroelectric devices in mainstream semiconductor technology. A breakthrough occurred in 2011 with the discovery of ferroelectricity in 10 nm-thick Si-doped HfO_2_ thin films, where top electrode (TE) capping followed by annealing and cooling induced interfacial stress and confinement effects that stabilized the metastable orthorhombic phase (Pca2_1_) responsible for ferroelectricity. Importantly, HfO_2_ had already been widely adopted in CMOS technology through atomic layer deposition (ALD), and this finding consequently sparked intense interest in ferroelectric memories owing to their CMOS compatibility and scalability toward advanced technology nodes [4–10]. Representative device types include the one-transistor-one-capacitor (1T-1C) ferroelectric random-access memory (FeRAM) [11], ferroelectric tunnel junction (FTJ) [12], ferroelectric field-effect transistor (FeFET) [13, 14], and ferroelectric memcapacitor (FeCAP) [15].

Fluorite-structured ferroelectrics have emerged as a promising material platform for ferroelectric memory devices. These materials are compatible with both silicon and a wide range of metal electrodes used in the semiconductor industry. Notably, HfO_2_ has long been employed as a high-k gate dielectric in CMOS transistors, while ZrO_2_, one of the most promising dopants for stabilizing ferroelectricity in HfO_2_, has been widely used in Dynamic random-access memory (DRAM) cell capacitors. Thanks to this strong compatibility with CMOS process technologies, functional devices including FeRAM and FeFET have already been demonstrated, while research efforts on FTJ and FeCAP devices are rapidly accelerating. This unique position at the intersection of well-established materials infrastructure and novel ferroelectric functionality has driven intense development in the field. Despite their promise, HfO_2_ based ferroelectrics face persistent challenges in reliability metrics such as endurance and retention, as well as in suppressing competing non-ferroelectric phases. Among these, the quality of the interface between the ferroelectric film and its surrounding layers is one of the most critical issues, which can strongly influence switching behavior and device stability. Materials and process optimization remain necessary to address these issues. Another major limitation is the restricted stabilization of the ferroelectric orthorhombic phase at very thin film thicknesses, which is essential for scaling to future device generations. Addressing these challenges requires an integrated approach encompassing materials design, interface engineering, and process refinement. Beyond their role in conventional non-volatile memory (NVM), HfO_2_-based ferroelectrics have been utilized for a variety of in-memory computing paradigms aimed at overcoming the limitations of traditional von Neumann architectures. Their intrinsic domain-switching behavior allows partial polarization states to be accessed, enabling multi-level conductance for neuromorphic computing. The stochastic distribution of initial ferroelectric domains can serve as a high-entropy physical source for hardware security primitives such as physically unclonable functions (PUFs). Furthermore, their non-volatility and fast switching make them suitable for associative memory architecture. These properties make HfO_2_-based ferroelectrics a key enabler for emerging computing applications, with growing research interest in both material and device-level optimization.

In this paper, we review the historical development of ferroelectric memories from a material–device integration perspective, with a particular focus on hafnia-based ferroelectrics. Section 2 traces the evolution of ferroelectric materials, from the discovery of ferroelectricity and early perovskite ferroelectrics to the recent emergence of hafnia-based fluorite ferroelectrics. Section 3 discusses HfO_2_-based ferroelectric memory architectures and their technological progress. Section 4 reviews applications of HfO_2_-based ferroelectric devices in in-memory computing, including neuromorphic computing, hardware security, and associative memory. Section 5 addresses the current challenges of ferroelectric memories and explores potential solutions. Finally, we provide a forward-looking perspective on future research strategies that integrate material innovations with device and architectural design for next-generation memory technologies.

## Ferroelectric materials

### Historical development of ferroelectric materials

The concept of ferroelectricity, characterized by two stable spontaneous polarization states, was first introduced by Erwin Schrödinger in 1912 [16]. It was experimentally demonstrated for the first time in 1921 by Valasek using Rochelle salt [17–19]. During the following decade, research on Rochelle salt continued, but its ferroelectric properties were found to be highly sensitive to environmental factors such as humidity. In 1935, Busch et al. discovered ferroelectricity in KH_2_PO_4_, which exhibited greater chemical stability and therefore stimulated further investigation [20]. However, its Curie temperature (*T*_c_) was lower than room temperature, which limited its practical use. In the early 1940s, during World War II, the perovskite-structured BaTiO_3_ was discovered. This material offered both room-temperature stability and a high dielectric constant (*ε*_r_ > 1000), marking a breakthrough that led to intensive research on perovskite-based ceramics and greatly advanced the understanding and application of ferroelectric phenomena [21–23]. Structurally, perovskite‐structured ferroelectrics possess the general chemical formula ABO_3_, where two cations of different sizes occupy the A‐site (cube corners) and B‐site (cube center), respectively. The B cation resides at the center of an octahedron formed by six oxygen atoms. Ferroelectricity arises when the B cation is slightly displaced from the octahedral center, generating an electric dipole, and when these dipoles align in a common direction, spontaneous polarization emerges. Following the discovery of BaTiO_3_, the Landau–Ginzburg–Devonshire (LGD) phenomenological model was developed to describe ferroelectricity and its phase transitions [24–27]. In the subsequent decades, extensive research was devoted to perovskite‐structured ferroelectrics, with one of the most significant findings being the Pb(Zr,Ti)O_3_ (PZT) solid solution. PZT exhibits a high *T*_c_ of approximately 400 °C and a large remanent polarization (*P*_r_) of 10–40 μC cm^−2^. Furthermore, by tuning the Zr/Ti ratio to form a morphotropic phase boundary, its piezoelectric properties are significantly enhanced, leading to major advancements in ferroelectric research [28–30]. In 1960, Cochran and Anderson independently proposed the concept of the soft mode, in which a specific phonon mode at the center of the Brillouin zone gradually softens with decreasing temperature, causing its frequency to approach zero. As a result, atoms remain in a displaced position rather than returning to their equilibrium sites, leading to the emergence of spontaneous polarization. This phenomenon established the fundamental basis for understanding displacive phase transitions in ferroelectrics [31–33].

**Fig. 1:**
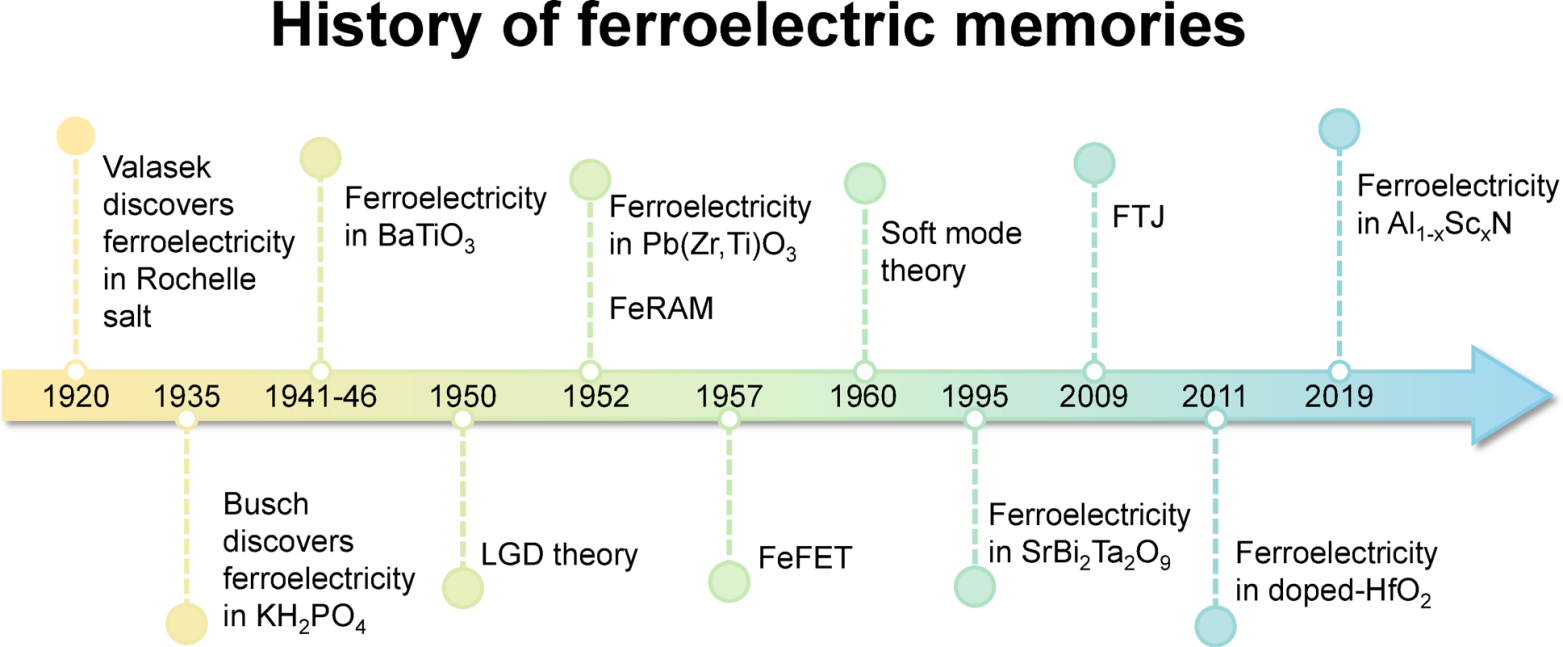
Historical development of ferroelectric (FE) materials and devices

Also, FeRAM and FeFET were first proposed in the 1950s [34] and 1960s [35], respectively. These devices demonstrated the potential to exploit the fast polarization switching speed and low power consumption of ferroelectric materials for memory applications, generating significant interest in their integration into memory and semiconductor devices. However, conventional perovskite ferroelectrics such as PZT and BaTiO_3_ exhibited severe fatigue issues in FeRAM. In 1995, the layered perovskite SrBi_2_Ta_2_O_9_ (SBT) was reported for the first time [36], offering high fatigue resistance and overcoming the lifetime limitations of PZT-based FeRAM, which accelerated its commercialization. In 2009, Garcia et al. reported the first FTJ based on BaTiO_3_ [37], introducing a new ferroelectric memory concept that employed a few-nanometer-thick ferroelectric barrier instead of the thick films used in FeRAM, enabling low-power, high-speed, non-volatile operation. In 2011, ferroelectricity was first observed in silicon-doped HfO_2_ thin films with a fluorite structure [38], marking a paradigm shift in ferroelectric research. Given that HfO_2_ was already widely used as a high-k dielectric in CMOS processes, its compatibility with existing semiconductor manufacturing and ability to retain ferroelectricity in ultra-thin films (< 10 nm) made it highly attractive for device scaling. This discovery triggered a surge of research into its integration in logic and memory devices. In 2019, Fichtner et al. reported ferroelectricity in Sc-doped AlN (Sc_x_Al_1−x_N) [39], exhibiting a *T*_c_ above 1000 °C and a *P*_r_ of ~ 110 µC/cm^2^, thereby introducing a high-temperature-stable ferroelectric material with potential to expand the application range of ferroelectric devices. Figure 1 summarizes the historical development of ferroelectric memories, while Table 1 presents the material properties of representative ferroelectrics, including the PZT, SBT, doped HfO_2_, and (Al,Sc)N.

**Table 1:** Material properties of representative ferroelectric materials, including Pb(Zr,Ti)O_3_, SrBi_2_Ta_2_O_9_, doped HfO_2_, and (Al, Sc)N

	Pb(Zr, Ti)O_3_	SrBi_2_Ta_2_O_9_	Doped HfO_2_	(Al, Sc)N
P_r_ [μC/cm^2^]	10–40	5–10	10–40	80–110
E_c_ [kV/cm]	50–70	30–50	800–2000	2000–5000
*ε*_r_	~ 400	~ 200	~ 30	~ 25
Min. thickness [nm]	50	–	< 5	< 10

### Hafnia-based ferroelectricity

**Fig. 2:**
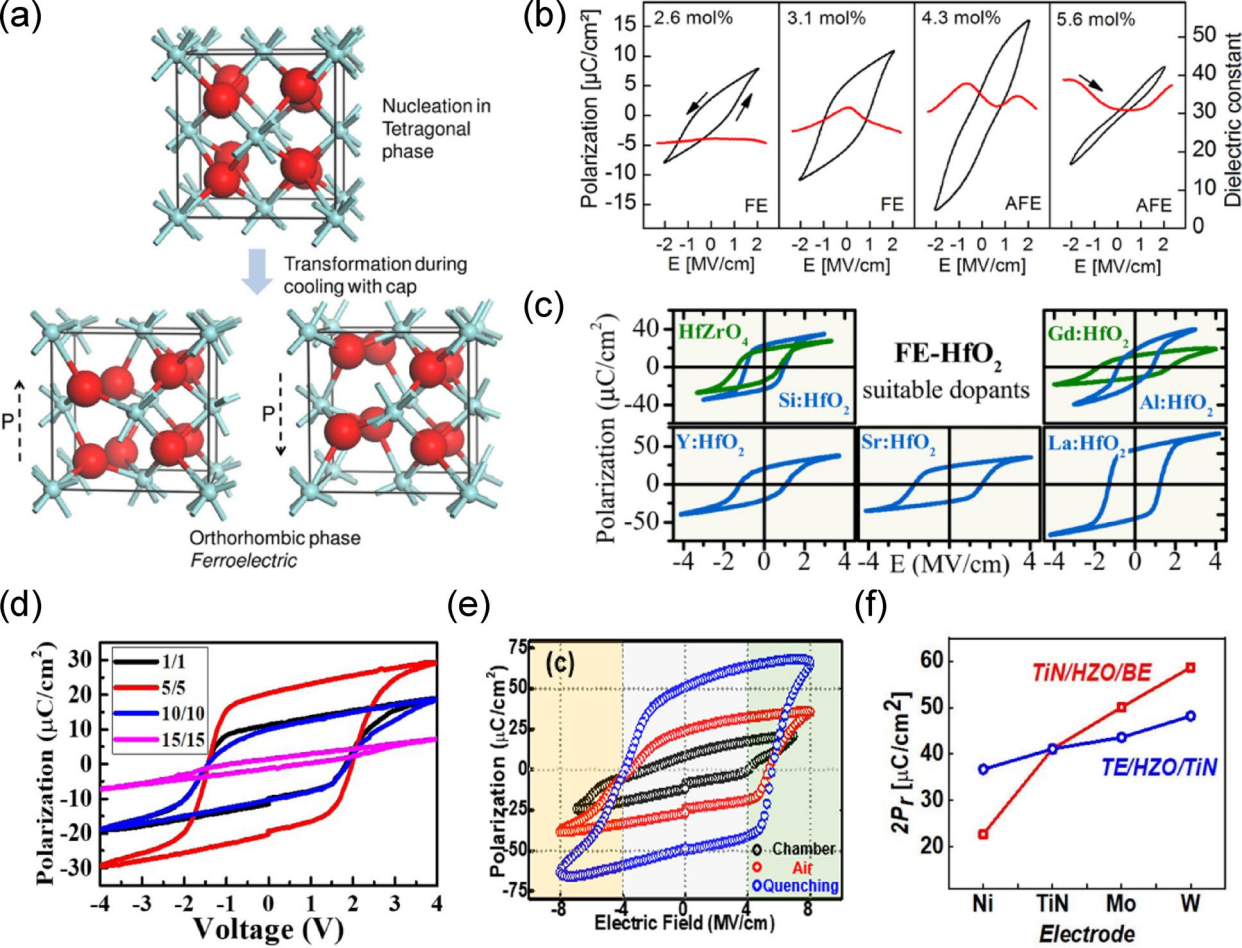
Hafnia-based ferroelectrics. **a** Schematic illustration of the phase transformation from the tetragonal to the orthorhombic phase in Si:HfO_2_ thin films, leading to the formation of a ferroelectric phase with two distinct polarization states (red atoms: oxygen, white atoms: hafnium). **b** *P*–*E* curves (black, left y-axis) and AC dielectric constant–electric field characteristics (red, right y-axis) for different SiO_2_ admixture amounts. As the silicon content increases, the polarization curves transition from FE to antiferroelectric (AFE), with the dielectric constant shifts from exhibiting a single peak to showing two distinct peaks. Reproduced with permission from Ref [38]. Copyright 2011, American Institute of Physics. **c** *P*–*E* curves of TiN/8–10 nm X:HfO_2_ or HfZrO_4_/TiN capacitors with various dopants X. Reproduced with permission from Ref [42]. Copyright 2013, IEEE. **d** *P*–*V* curves of TiN/Zr-doped HfO_2_ (HZO)/TiN capacitors for different ALD cycle ratios of HZO deposition. Reproduced with permission from Ref [53]. Copyright 2019 IEEE. **e** *P*–*E* characteristics of Al:HfO_2_ ferroelectric capacitors for different cooling rates. Reproduced with permission from Ref [56]. Copyright 2020 IEEE. **f** Comparison of remanent polarization for HZO ferroelectric capacitors with different metal electrodes. Reproduced with permission from Ref [59]. Copyright 2021, IEEE

As discussed earlier, Böscke et al. first reported ferroelectricity in Si-doped HfO_2_ thin films, which initiated a new research direction in hafnia-based ferroelectrics [38]. This breakthrough attracted significant interest, as HfO_2_ was already widely used as a high-k gate dielectric in CMOS technology thanks to the ALD deposition. Its compatibility with conventional semiconductor fabrication processes, ability to maintain ferroelectricity even in ultrathin (< 10 nm) films, and advantages for device scaling established hafnia-based ferroelectrics as promising candidates for next-generation nonvolatile memory and logic applications. In bulk at room temperature, HfO_2_ is monoclinic and non-ferroelectric, transforming to tetragonal and cubic phases at elevated temperatures. During deposition and thermal treatment, a high-temperature tetragonal phase may form and, upon cooling, particularly under a TE capping effect, transform into the non-centrosymmetric orthorhombic phase with switchable polarization. Figure 2a illustrates the tetragonal to orthorhombic phase transformation in Si:HfO_2_ thin films, yielding two polarization states (red: oxygen; white: hafnium). Figure 2b shows polarization–electric field (*P*–*E*) curves and AC dielectric constant–electric field characteristics for different Si contents, indicating that ferroelectric properties vary with dopant content [38]. In particular, as the silicon content increases, the polarization gradually shifts from FE to antiferroelectric (AFE), which is also evidenced by the dielectric constant exhibiting a single peak in the FE state and two peaks in the AFE state. Here, *P*_r_, defined as the magnitude of the spontaneous polarization that remains after the external electric field is removed from the ferroelectric layer, reflects the strength of the ferroelectric behavior. A large *P*_r_ not only signifies strong ferroelectricity but also enhances the charge-sensing signal, read current margin, and on/off ratio in FeRAM, FeFET, and FTJ devices. Consequently, research on hafnia-based ferroelectrics has focused not only on understanding their ferroelectric characteristics but also on achieving higher *P*_r_ values.

Hafnia-based ferroelectrics with various dopants (e.g., Zr, Gd, Y, La) have been reported to stabilize the orthorhombic phase [40–45]. Figure 2c compares *P*–*E* curves of TiN/8–10 nm X:HfO_2_ or HfZrO_4_/TiN capacitors with different dopants X and notably, the La-doped HfO_2_ thin film exhibited the *P*_r_, reaching approximately 45 μC/cm^2^ (Müller et al. [42]). It has also been reported that adjusting the dopant concentration influences the ferroelectric properties [46]. Furthermore, hafnia-based ferroelectric films have been shown to retain ferroelectricity at thicknesses down few nanometers [47]. In Zr-doped HfO_2_, particularly Hf_0.5_Zr_0.5_O_2_ (HZO) films, a counterintuitive enhancement of polarization has been observed when the thickness was reduced to ultra-thin regime of 1 nm [48]. This finding shows that the enhancement effect persists down to the thickness of at least two fluorite-structure unit cells, overcoming the deleterious depolarization field that typically arises in the ultrathin regime of conventional perovskite films [49]. By mitigating this scaling limitation, it demonstrates the potential for realizing next-generation, ultra-scaled ferroelectric transistors. While many doped HfO_2_ films require high-temperature annealing, which limits the integration of ferroelectric circuits into back-end-of-line (BEOL) processes, HZO has shown ferroelectricity within a low thermal budget (400–450 °C), including cases where crystallization occurs during TE TiN ALD without an additional heat treatment [50, 51]. Kim et al. further showed that optimizing the TiN TE thickness in TiN/HZO/TiN capacitors enabled high 2*P*_r_ ~ 45 μC/cm^2^ at 400 °C [52].

Also, several studies have been conducted to achieve higher *P*_r_ values by optimizing the ferroelectric layer through deposition methods, annealing conditions, and metal electrode engineering. Liao et al. engineered the grain size of HZO thin films by optimizing the HfO_2_-to-ZrO_2_ cycle ratio during ALD, achieving a 2*P*_r_ value of 41 μC/cm^2^. The corresponding polarization–voltage (*P*–*V)* curves of TiN/HZO/TiN capacitors for different ALD cycle ratios are shown in Fig. 2d [53]. Peng et al. demonstrated that employing a superlattice HZO thin film in metal–ferroelectric–metal (MFM) capacitors increased *P*_r_ compared to conventional HZO films, while simultaneously reducing leakage current and improving endurance, achieving over > 10^12^ switching cycles [54]. Zhou et al. investigated the effect of annealing temperature on Al-doped HfO_2_ (HAO) thin films, varying the temperature from 600 to 800 °C. By optimizing the annealing temperature to 800 °C, they engineered the crystallization of the HAO film and achieved a high *P*_r_ value of 23.7 μC/cm^2^ [55]. Ku et al. analyzed the influence of post-cooling processes after annealing the HAO ferroelectric layer, comparing chamber cooling, air cooling, and rapid quenching in deionized water. The corresponding *P*–*E* curves for each cooling condition are shown in Fig. 2e [56]. They found that rapid quenching induced greater stress/strain in the HAO films, which promoted the stabilization of the orthorhombic phase. As a result, a drastic increase in *P*_r_ was observed, with 2*P*_r_ values approaching ~ 100 μC/cm^2^.

Hoffmann et al. compared Gd-doped HfO_2_ ferroelectric thin films using TiN and TaN electrodes. When TaN was used, a higher *P*_r_ of 35 μC/cm^2^ was obtained compared to TiN. This enhancement was attributed to the fact that, during thermal treatment, the TaN electrode underwent more extensive interfacial oxidation than TiN, which increased the concentration of oxygen vacancies in the HfO_2_ layer. The increased vacancy concentration lowered the total energy of the orthorhombic phase in the lattice, thereby stabilizing the ferroelectric phase and enabling a higher *P*_r_ [41]. Goh et al. investigated the ferroelectric properties of a 4.5 nm HZO film with a TiN TE using W, Mo, Pt, TiN, and Ni as bottom electrodes (BEs). Among these, the W yielded the highest value, with a 2*P*_r_ of 62.4 μC/cm^2^ [57]. Similarly, Lee et al. examined the impact of various electrode materials for both the top and bottom contacts in HZO film. As shown in Fig. 2f, W electrodes produced the highest *P*_r_ values for both configurations compared to other electrodes. This enhancement was because of the lower coefficient of thermal expansion (CTE) of tungsten, which induced greater tensile stress in the HZO film during annealing, thereby increasing the orthorhombic phase fraction and improving ferroelectric properties [58].

## Ferroelectric-based memory devices

### Ferroelectric random-access memory (FeRAM)

**Fig. 3:**
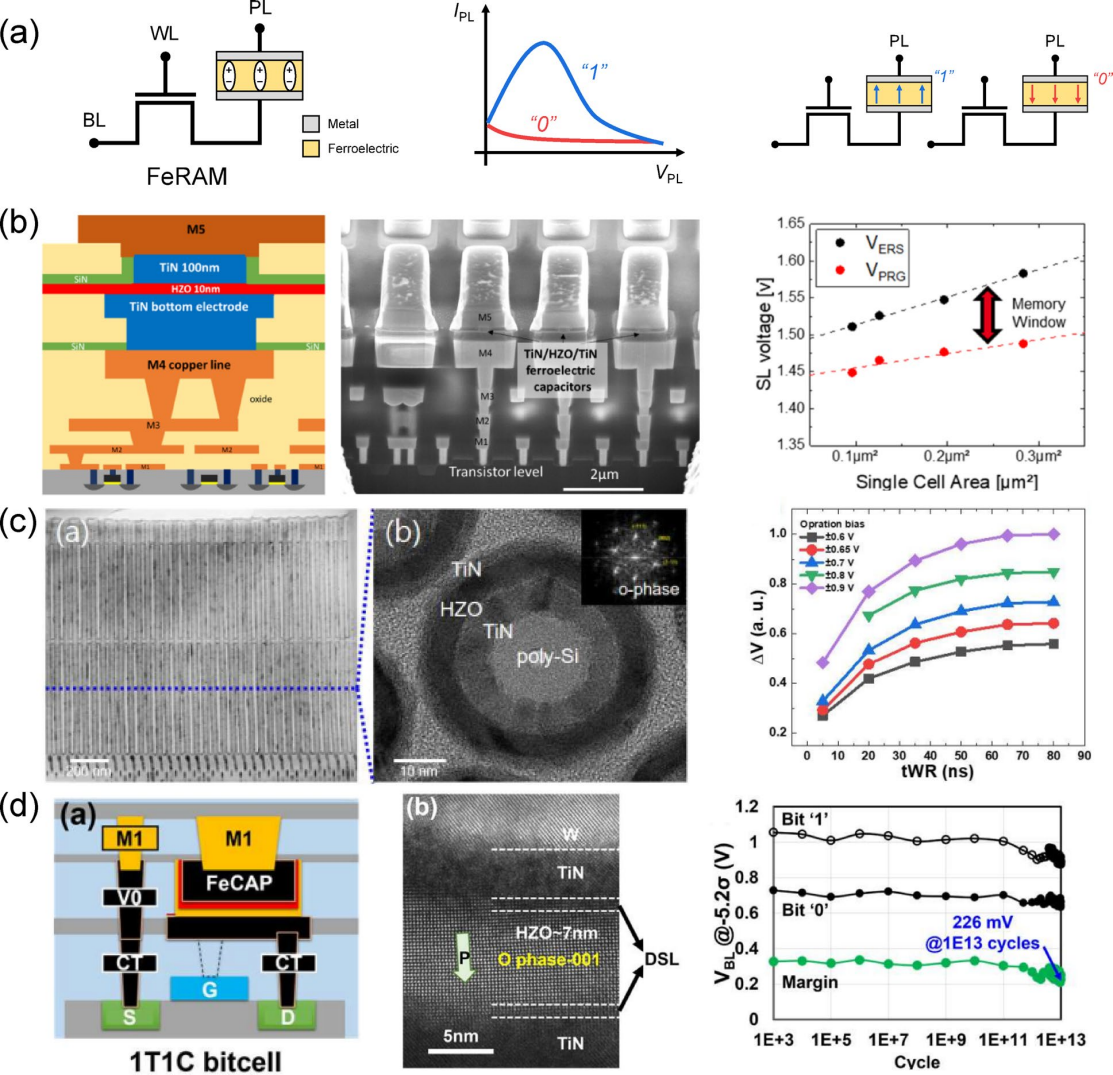
FeRAM. **a** Schematics of 1T-1C FeRAM structure with *I*_PL_–*V*_PL_ read characteristics and two polarization states. **b** Schematic of BEOL-compatible FeRAM cell co-integrated with 130 nm CMOS and fabricated with TiN/HZO/TiN ferroelectric capacitors (left panel), cross-sectional SEM image showing the capacitor stack integrated above the transistor level (middle panel), and measured source line voltage versus single-cell area demonstrating the memory window scaling (right panel). Reproduced with permission from Ref [63]. Copyright 2019, IEEE. **c** Cross-sectional TEM image of a 1T-1C FeRAM with ultra-thin 5 nm HZO (left panel), high-resolution TEM and FFT image showing orthorhombic phase stabilization in TiN/HZO/TiN/poly-Si structure (middle panel), and measured ΔV as a function of write pulse width (*t*_WR_) for various operation voltages (right panel). Reproduced with permission from Ref [66]. Copyright 2021, IEEE. **d** Schematic of a 1T-1C FeCAP bitcell for 3D integration (left panel), cross-sectional high-resolution TEM image showing a 7 nm HZO layer with orthorhombic phase orientation in the TiN/HZO/TiN stack (middle panel), and measured sensing voltage margin as a function of endurance cycles at 125 °C (> 1E13 cycles) (right panel). Specifically, two defect shielding layers (DSLs) are deposited on both sides of the HZO film to eliminate excess charged defects at the interfaces, thereby suppressing leakage and improving endurance. Reproduced with permission from Ref [70]. Copyright 2025, IEEE

FeRAM is a non-volatile memory based on a 1T-1C architecture, in which the dielectric layer of the capacitor is replaced with a ferroelectric material. Figure 3a shows the schematic of a FeRAM cell consisting of an access transistor and a FeCAP. During a write operation, the word line (WL) is activated to turn on the transistor, and a voltage is applied between the plate line (PL) and bit line (BL) to set the polarization direction of the ferroelectric layer. For readout, a voltage pulse is applied to the PL, inducing a polarization reversal charge at the BL according to the stored polarization state. The resulting signal, which differs depending on the polarization direction, is sensed by the sense amplifier (SA) to distinguish between logic “0” and “1”. Since polarization switching occurs during reading, FeRAM exhibits a destructive-read characteristic, requiring a write-back process after each read operation. The concept of FeRAM was first proposed in the 1950s [34], and with the maturation of planar ferroelectric PZT thin-film processing, commercial products emerged in the early 1990s for smart cards, consumer electronics, and industrial control systems [59]. However, perovskite-based FeRAM required relatively large cell structures to ensure sufficient capacitance, and its thin-film fabrication process posed integration challenges, confining its use to low-density, application-specific markets [60].

The introduction of CMOS-compatible HfO_2_-based ferroelectrics revitalized FeRAM scaling by enabling three-dimensional (3D) capacitor integration. In 2014, a deep-trench capacitor structure utilizing Al-doped HfO_2_ demonstrated the potential for 3D-integrated non-volatile memory [61]. This progress advanced further in 2019, when Francois et al*.* demonstrated that a TiN/HZO/TiN ferroelectric capacitor could be monolithically integrated into the BEOL of a 130 nm CMOS process, marking a significant advance in embedded FeRAM scalability [62]. As shown in Fig. 3b (left panel), the cross-sectional schematic highlights the placement of ferroelectric capacitors between metal interconnect layers (M4 and M5), while the SEM image (middle panel) confirms successful fabrication of nanoscale capacitors down to 300 nm. Also, the electrical characterization (right panel) reveals stable memory windows as the cell area is scaled, with erase and program voltages maintaining clear separation even at sub-1µm^2^ dimensions. Overall, these results demonstrate that BEOL-compatible HZO-based FeCAPs can achieve aggressive cell scaling while preserving robust ferroelectric properties, enabling high-density, low-power embedded non-volatile memory without additional process complexity.

Subsequently, several Hf_0.5_Zr_0.5_O_2_-based 1 T-1C FeRAM arrays have been demonstrated, offering low-voltage operation and high endurance [63, 64]. Nevertheless, when scaled down to ultra-thin films, FeRAM characteristics are prone to degradation due to depolarization fields, interfacial dead layers, and stabilization of competing non-ferroelectric phases. In 2021, Sung et al*.* reported an ultra-thin HZO study that directly addressed these limitations [65]. As shown in the left panel of Fig. 3c, a cross-sectional image reveals the vertical storage node structure with a 1z nm MFM capacitor. The middle panel of Fig. 3c presents High-resolution transmission electron microscopy (HR-TEM) and Fast Fourier transform (FFT) patterns confirming the stabilization of the orthorhombic phase in a TiN/HZO (5 nm)/TiN/poly-Si stack. The right panel of Fig. 3c plots the dependence of ΔV on operating bias and write-pulse width, demonstrating that even at low voltages, a sufficient memory window can be maintained by shortening the pulse width. Overall, these results prove that reliable ferroelectric switching and fast write operations are achievable in 5 nm-thick HZO, supporting the feasibility of ultra-low-voltage, high-speed FeRAM scaling for future applications. In 2022, Toprasertpong et al. highlighted further reduced operating voltage, improved breakdown tolerance, and endurance exceeding 10^12^ cycles for HZO-based FeRAM [66], while Haratipour et al. showcased a 28 nm-class, ultra-high-density, high-speed FeRAM targeting next-generation embedded memory [67]. In parallel, a BEOL-integrated 16 kb HfO_2_:Si FeRAM array achieved 4 ns programming, 10^7^-cycle endurance, 125 °C retention, and solder-reflow compatibility [68].

Most recently, a 3D trench-type 1 T-1C FeRAM integrating a 7 nm-thick Hf_0.5_Zr_0.5_O_2_ layer with dual-side defect shielding layers (DSLs) has been demonstrated, achieving significant reliability improvements for embedded non-volatile memory applications [69]. As shown in the left panel of Fig. 3d, the cross-sectional schematic illustrates the vertical trench capacitor integrated above the access transistor, with DSLs placed at both the top and bottom interfaces of the ferroelectric layer to suppress oxygen vacancy migration and interface defect formation. The middle panel of Fig. 3d presents a high-resolution TEM image clearly showing the orthorhombic-phase HZO film sandwiched between TiN electrodes, where the DSLs appear as a distinct interfacial layer, confirming effective defect control. The right panel of Fig. 3d shows endurance and retention characteristics, where the memory window remains above 200 mV even after 10^11^ write and 10^13^ read cycles followed by 125 °C baking, and retention extrapolation indicates > 10 years of stability at 125 °C. These results demonstrate that the defect-engineered trench-type FeRAM achieves exceptional endurance, thermal stability, and array-level reliability, meeting the stringent requirements to replace eFlash in industrial eNVM applications.

### Ferroelectric tunnel junction (FTJ)

**Fig. 4:**
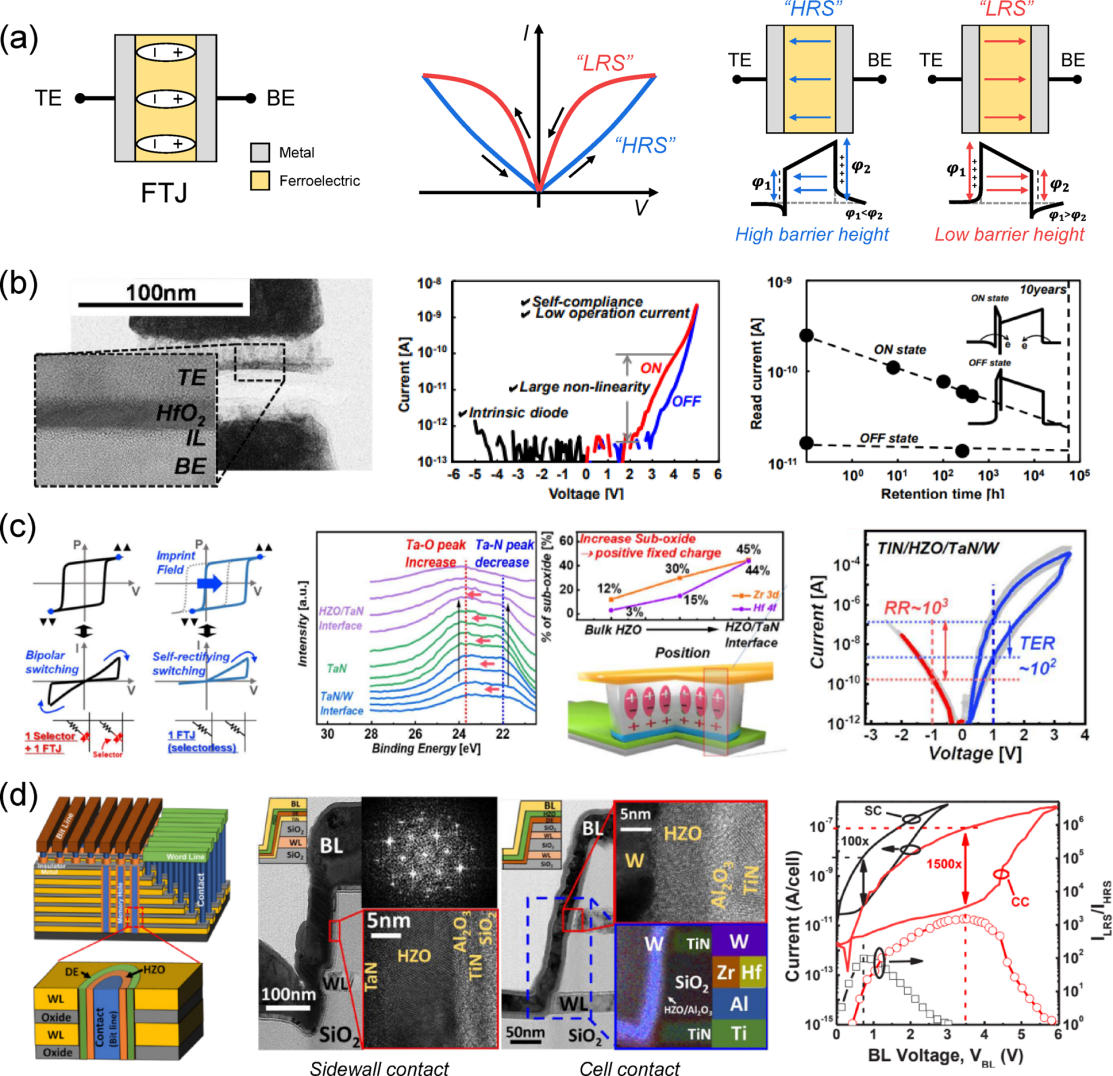
FTJ. **a** Schematics of MFM FTJ with *I*–*V* characteristics for high/low resistance states and corresponding resistance changes due to barrier height variation. **b** Cross-sectional TEM image of an HfO_2_-based resistive switch showing a FTJ with intrinsic diode property (left panel), schematic illustration of current–voltage characteristics demonstrating low operation current and self-rectification (middle panel), and retention characteristics over time for different states (right panel). Reproduced with permission from Ref [73]. Copyright 2016, IEEE. **c** Comparison of switching characteristics between conventional bipolar switching and self-rectifying switching in HZO-based FTJs (left panel), highlighting the imprint field effect. X-ray photoelectron spectroscopy (XPS) depth profile for Ta 4f, the relative sub-oxide portions of HfO_2_ and ZrO_2_ obtained from the Hf 4f and Zr 3d XPS spectra across the bulk HZO to the HZO/TaN interface, and a schematic of the TiN/HZO/TaN/W stack (middle panel), implying that V_o_^++^, which induces the imprint field, is generated as the TaN layer reacts with the HZO layer and contributes to the built-in potential responsible for self-rectification. The electrical performance of the TiN/HZO/TaN/W FTJ (right panel) exhibits a high rectification ratio of ~ 10^3^ and a large TER of ~ 10^2^. Reproduced with permission from Ref [84]. Copyright 2021, IEEE. **d** Schematic illustration of the vertical FTJ (V-FTJ) architecture, where the ferroelectric HZO layer is conformally deposited along the sidewall of a vertical hole (left panel). Cross-sectional TEM images of the sidewall contact (SC) and cell contact (CC) structures are shown, respectively, while the FFT image demonstrates the crystallinity of the HZO and the EDX analysis verifies the fabricated structure and elemental composition (middle panel). Electrical measurements of the *I*–*V* curves and *I*_LRS_/*I*_HRS_ ratios for SC and CC structures, demonstrating a high on/off ratio of 1500 × for CC and a self-rectifying ratio of 100 × for SC, thereby enabling selectorless operation in 3D architectures (right panel). Reproduced with permission from Ref [85]. Copyright 2023, IEEE

The FTJ is a two-terminal device in which a ferroelectric thin film is sandwiched between TE and BE, optionally with an additional insulating or semiconducting interlayer (IL), as shown in Fig. 4a. Current transport occurs via quantum tunneling, and the polarization direction of the ferroelectric layer modulates the interfacial charge distribution at the electrode/ferroelectric interface. This modulation changes both the height and the width of the tunneling barrier, enabling two distinct resistance states within the same structure, a low-resistance state (LRS) and a high-resistance state (HRS). As shown in the right panel of Fig. 4a, when the polarization points toward the TE, the barrier height on the BE side increases relative to that on the TE side, and the effective tunneling barrier becomes wider, which lowers the tunneling probability and results in the HRS. Conversely, when the polarization points toward the BE, the barrier height on the BE side decreases relative to that on the TE side, and the effective tunneling barrier becomes narrower compared with the HRS, leading to the LRS. This binary distinction in resistance can be exploited for non-volatile memory applications. Because FTJs utilize polarization-controlled tunneling current, they are inherently non-volatile, offer excellent scalability to the ultrathin regime, and operate at low energy. Also, their two-terminal configuration is highly compatible with crossbar array architectures, making FTJs promising candidates for high-density non-volatile memories and in-memory computing devices. At the microscopic level, the polarization of the ferroelectric layer induces bound charges at the interfaces, which interact with the finite screening length of the electrodes, asymmetry in metal work functions, and interfacial dipoles to modulate the electrostatic potential barrier. Since the tunneling probability depends exponentially on both barrier height and width, polarization reversal alone can induce a substantial change in the tunneling current.

Although polarization-controlled tunneling was first reported by Esaki et al*.* in 1971 [70], the first solid-state FTJs were not realized until 2012 [71], mainly due to the difficulty of fabricating high-quality ferroelectric films only a few nanometers thick with pristine interfaces. This challenge was addressed by advances in ALD and the discovery of ferroelectricity in hafnia-based films [38, 40], enabling CMOS-compatible FTJ fabrication and driving rapid progress in device research and applications. In 2016, Fujii et al*.* demonstrated the first hafnia-based FTJ using a TiN/Si:HfO_2_ (~ 5 nm)/TiN stack [72]. The left panel of Fig. 4b presents a cross-sectional TEM image revealing the device structure, where the ferroelectric HfO_2_ layer and the IL are clearly identified between the TE and BE. The middle panel of Fig. 4b shows the current–voltage (*I*–*V*) characteristics, indicating a distinct conductance change upon polarization reversal due to modulation of the tunneling barrier. Notably, the device exhibited a self-rectifying characteristic without an external selector, which is a key advantage for direct crossbar integration. The right panel of Fig. 4b shows that the on-current gradually decreases over time, possibly due to electron trapping during retention. Further improvement of on-state stability could be achieved by developing FE-HfO_2_ films with fewer defects, thereby suppressing electron trapping.

Following this, FTJ designs have evolved into four primary structures: MFM, metal–ferroelectric–insulator–metal (MFIM), metal–ferroelectric–semiconductor (MFS), and metal–ferroelectric–insulator–semiconductor (MFIS). In MFM and MFS devices, polarization reversal modifies the average barrier height, producing the tunneling electroresistance (TER) effect. The TER magnitude depends on the remanent polarization and the difference in electrode screening lengths [73–77]. Ambriz-Vargas et al. modeled the *I*–*V* characteristics of an MFM FTJ using a direct tunneling approach, extracting potential barriers of 1.86/2.36 eV (ON state) and 2.75/2.22 eV (OFF state) [73]. While MFM structures generally show modest TER due to small screening-length asymmetry, MFS designs can significantly enhance TER by altering semiconductor carrier concentration via polarization control, enabling accumulation, inversion, or depletion. For example, Jiao et al. achieved a TER of ~ 800 using an MFS FTJ with a Nb-doped SrTiO_3_ electrode and a 5.8 nm-thick polycrystalline HZO layer [74]. MFIM and MFIS structures insert an insulating layer to decouple the ferroelectric film from direct electrode contact, which reduces interface defects, improves endurance, and increases TER via barrier asymmetry [78–82]. However, the IL also introduces additional depolarization fields that can degrade retention, making IL thickness optimization essential for balancing endurance, TER, and retention.

Recent work has also utilized asymmetric internal fields for self-rectifying FTJs. The left panel of Fig. 4c schematically shows *P*–*V* loops and *I*–*V* characteristics, illustrating how inserting a TaN diffusion-barrier layer into a TiN/HZO/TaN/W stack generates a positive imprint field that shifts the *P*–*V* loop and enables self-rectifying switching without an external selector [83]. The middle panel of Fig. 4c presents the X-ray photoelectron spectroscopy (XPS) depth profile for Ta 4f, the relative sub-oxide portions of HfO_2_ and ZrO_2_ obtained from the Hf 4f and Zr 3d XPS spectra across the bulk HZO to the HZO/TaN interface, and a schematic of the TiN/HZO/TaN/W stack. The XPS analysis shows that, compared with the TaN/W interface, the HZO/TaN interface exhibits an increased Ta–O bonding peak and a decreased Ta–N bonding peak, implying that the TaN layer absorbs oxygen from the HZO layer and oxidizes into Ta_x_O_y_. In addition, the proportion of sub-oxides increases sharply toward the HZO/TaN interface, indicating that the TaN electrode reacts with the HZO layer and forms V_o_^++^ at the interface, which in turn leads to the imprint effect. In the right panel of Fig. 4c, the devices exhibit pronounced diode-like *I*–*V* asymmetry, with a rectifying ratio of ~ 10^3^ at a read voltage of ~ 1 V and a TER of ~ 10^2^, enabling stable read operation in crossbar arrays projected up to N ≈ 4 k, significantly higher than the N ≈ 30 limit for a non-rectifying TiN/HZO/W control device.

In parallel, vertical FTJ integration has been studied to overcome the footprint limitations of planar cells [84, 85]. Vertical architecture enables extreme integration density but has challenges such as conformal deposition of ultrathin ferroelectrics on vertical sidewalls, interface preservation, and low-resistance electrode formation. Lee et al. demonstrated a NAND/NOR-based 3D-stacking vertical AFE FTJ array, as illustrated in the left panel of Fig. 4d [84]. In particular, they fabricated two types of 3D vertical structures, sidewall contact (SC) and cell contact (CC), and the middle panel of Fig. 4d presents cross-sectional TEM images of the two fabricated arrays. The memory holes were formed with a side length of 1 μm, and the SC structure was distinguished by the deposition of TiN on the sidewalls. Both AFE FTJ arrays were fabricated using HZO (Zr ≈ 90%) with an Al_2_O_3_ interlayer, deposited on the memory-hole sidewalls by ALD and subsequently crystallized by rapid thermal anneal (RTA). The FFT image in the middle panel of Fig. 4d confirms the crystallinity of the HZO formed on the memory-hole sidewalls, while energy-dispersive X-ray (EDX) analysis shows the elemental mapping of the fabricated arrays. Furthermore, first-order reversal curve (FORC) analysis revealed a built-in bias originating from fixed charges in the Al_2_O_3_ and interfacial dipoles, which resulted in a nonzero remanent polarization at 0 V and contributed to the self-rectifying behavior. The right panel of Fig. 4d presents the *I*–*V* characteristics and the *I*_LRS_/*I*_HRS_ ratio for both SC and CC structures (line: current (A/cell); symbol: *I*_LRS_/*I*_HRS_; black: SC; red: CC). The CC suppressed HRS leakage through localized HZO crystallization and a smaller effective contact area, achieving an *I*_LRS_/*I*_HRS_ ratio of ~ 1500 × (versus ~ 100 × for SC) and an on-state current of ~ 100 nA per cell at a current density of ~ 83 A cm^−2^, sufficient for direct sensing by peripheral circuitry. The AFE FTJ also exhibited a rectification ratio > 1000 × , supported multilevel conductance tuning via erase-voltage adjustment, and demonstrated endurance exceeding 10^9^ program/erase cycles and retention beyond 10^4^ s, with extrapolated 10-year on/off ratios above 10 × . Selector-free read margins were projected to scale up to ~ 10^4^ crossbar lines in the CC configuration. These results highlight that optimized vertical antiferroelectric FTJ structures can combine high on/off ratios, strong rectification, and robust reliability, making them viable candidates for high-density BEOL-integrated non-volatile memories.

### Ferroelectric field-effect transistor (FeFET)

**Fig. 5:**
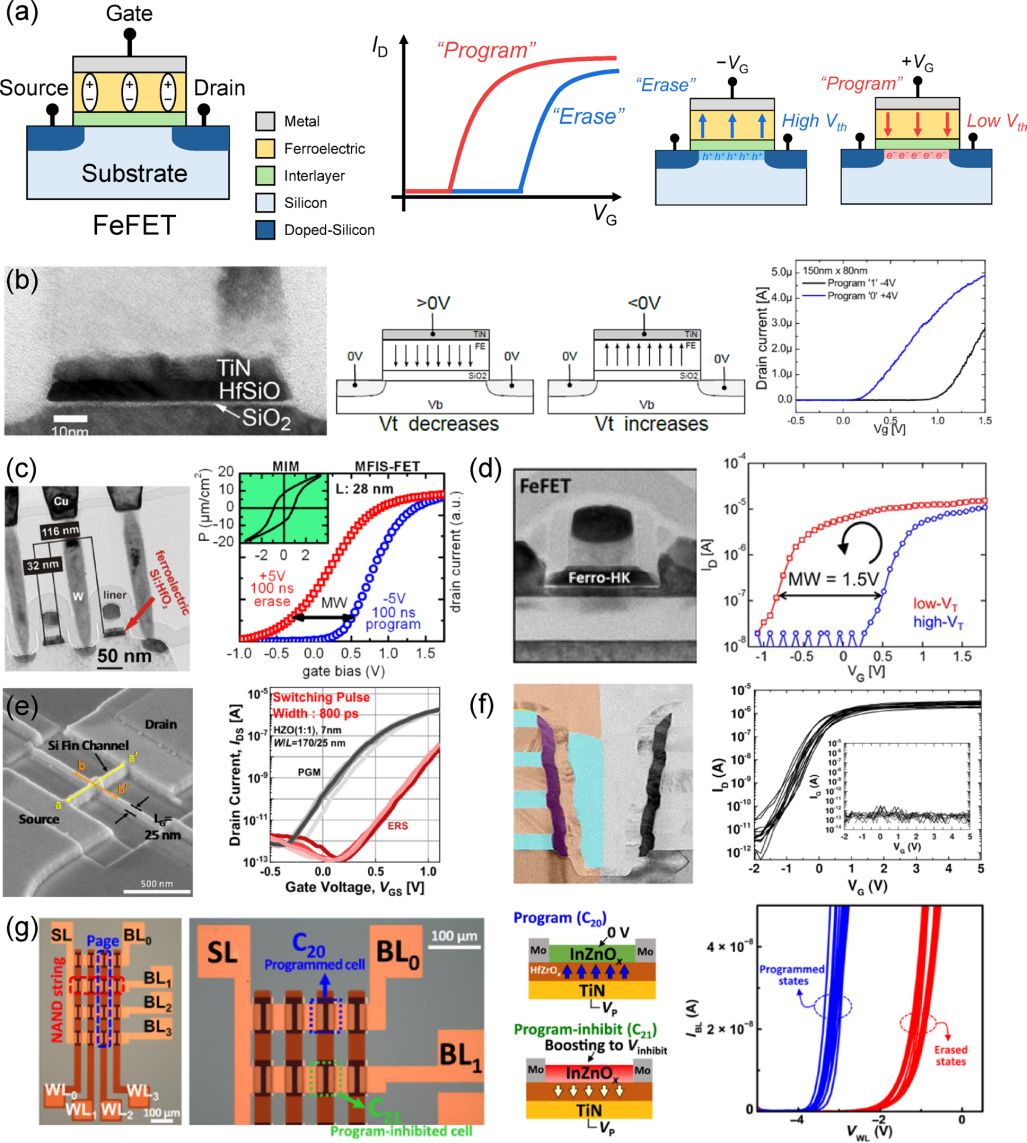
FeFET. **a** Schematics of MFIS FeFET with *I*_d_–*V*_g_ transfer curves and *V*_th_ shifts. **b** First reported hafnium oxide-based FeFET. Reproduced with permission from Ref [89]. Copyright 2011, IEEE. **c** 28 nm HKMG FeFET using Si:HfO_2_ thin films. Reproduced with permission from Ref [90]. Copyright 2012, IEEE. **d** FeFET fabricated with 22 nm FDSOI CMOS technology. Reproduced with permission from Ref [91]. Copyright 2017, IEEE. **e** FinFET-type FeFET using HZO thin films and a 25 nm technology node. Reproduced with permission from Ref [92]. Copyright 2020, IEEE. **f** Vertical FeFET NAND with a 3D macaroni-NAND architecture. Reproduced with permission from Ref [94]. Copyright 2018, IEEE. **g** FeFET NAND with an HZO thin film and an InZnO_x_ oxide semiconductor channel. Reproduced with permission from Ref [100]. Copyright 2021, American Assocation for the Advancement of Science

A FeFET is a three-terminal transistor-type non-volatile memory device that incorporates a ferroelectric layer as the gate dielectric as shown in Fig. 5a. The polarization state of the ferroelectric layer modulates the electric field applied to the channel, thereby shifting the threshold voltage (*V*_th_) and creating a memory window. Applying a positive gate voltage reduces *V*_th_, corresponding to the program state, whereas applying a negative gate voltage increases *V*_th_, corresponding to the erase state. The resulting *V*_th_ shift is opposite to that observed in conventional flash memories. In some cases, however, the program and erase states are defined following the flash memory convention, where the high-*V*_th_​ state is labeled as program and the low-*V*_th_ ​state as erase. While this reflects a difference in how the memory states are expressed, the relationship between the polarity of the applied write voltage and the direction of the *V*_th_ ​shift in FeFETs, which originates from the polarization of the ferroelectric layer, remains unchanged. Furthermore, compared with conventional flash memories, which perform program and erase operations via Fowler–Nordheim (FN) tunneling and therefore require high voltages and long programming times [86, 87], FeFETs enable low-power operation owing to their low operating voltage and fast switching speed.

In 2011, Böscke et al. first demonstrated a CMOS-compatible FeFET utilizing a Si:HfO_2_ ferroelectric thin film [88]. The left panel of Fig. 5b shows a cross-sectional TEM image of the gate stack of the fabricated device. The device incorporated an 8.5 nm ferroelectric layer and annealed at 1000 °C, resulting in the formation of a 1.2 nm SiO_2_ interlayer. The right panel of Fig. 5b shows the *I*_d_–*V*_g_ transfer curve of a short-channel device with dimensions of 150 nm × 80 nm, achieving a memory window of up to 1 V. In addition, non-volatile data retention for 10 years was demonstrated. Müller et al. fabricated a Si:HfO_2_-based FeFET using 28 nm high-k metal gate (HKMG) technology, as shown in the left panel of Fig. 5c [89]. Instead of conventional PZT ferroelectric, a hafnium-based ferroelectric material with high process scalability was employed, enabling the use of a 28 nm technology node with a height-to-length ratio of approximately 1. The right panel of Fig. 5c shows a memory window of 0.9 V, achieved using a ± 5 V program/erase voltage and a pulse width of 100 ns. Dünkel et al. reported a FeFET fabricated using 22 nm fully-depleted silcon on insulator (FDSOI) CMOS technology, scaled to a cell area of 0.025 μm^2^, as shown in the left panel of Fig. 5d [90]. This work demonstrated that FeFET memory can follow the ongoing scaling trend of logic technology while also proving scalability down to the 12 nm technology node. The right panel of Fig. 5d shows a corresponding *I*_d_–*V*_g_ transfer curve with a memory window of 1.5 V. Furthermore, write and read operations of a 32 Mbit memory array were demonstrated, highlighting the potential for low-cost, low-power expansion into Internet of Things (IoT) applications. Bae et al. demonstrated a ferroelectric FinFET with an HZO ferroelectric layer at the 25 nm technology node, as shown in the left panel of Fig. 5e [91]. The right panel of Fig. 5e shows the corresponding *I*_d_–*V*_g_ transfer curve, obtained under sub-nanosecond switching pulses, exceeding the nanosecond-level operation speeds reported in previous studies. This performance demonstrates the potential of this device as a memory candidate within the memory hierarchy for CPUs or cache memory, where extremely high performance is required.

Beyond scaling-focused research for logic devices, significant efforts have also been directed toward applying ferroelectric memory technology to high-density NAND Flash, referred to as ferroelectric NAND (FeNAND), leveraging its high process compatibility [92–94]. Florent et al*.* demonstrated a 3D macaroni NAND architecture integrating a Si-doped HfO_2_ ferroelectric layer as the memory layer, as shown in the left panel of Fig. 5f [93]. The right panel of Fig. 5f shows the corresponding *I*_d_–*V*_g_ transfer curve of the control gate transistor, measured by applying a 3 V pass bias to both the top and bottom selectors. The vertically stacked 3D FeFET structure exhibited a memory window of approximately 2 V, demonstrating the potential of ferroelectric memory for application in high-density NAND arrays. However, the MFS configuration of the FeFET structure inherently leads to the formation of an IL between the ferroelectric layer and the semiconductor channel during the annealing process. Because the relative permittivity of the SiO_2_ (~ 3.9) is significantly lower than that of the HfO_2_ based ferroelectric layer (~ 20–40), a substantial portion of the applied gate voltage drops across the IL, thereby reducing the electric field applied to the ferroelectric layer. This field reduction limits polarization switching and narrows the memory window, while the high electric field across the IL induces charge trapping, which further decreases the memory window and accelerates endurance degradation due to premature dielectric breakdown. To overcome these limitations, Ni et al. replaced the conventional SiO_2_ IL with high-k materials such as Al_2_O_3_, HfO_2_, and La_2_O_3_, and analyzed the correlation between the memory window and the IL material and thickness [95]. Their results demonstrated that an optimized IL improves electric field coupling to the ferroelectric layer, thereby widening the memory window, and positively influences both retention and endurance characteristics. Xiao et al. demonstrated an HZO-based FeFET in which a SiO_2_ IL was formed, followed by the deposition of a 1.5 nm ZrO_2_ seed layer prior to HZO deposition [96, 97]. This approach enhanced the ferroelectric properties of the HZO film, increasing the remanent polarization and coercive voltage, improving the interface characteristics between the ferroelectric layer and the IL, and reducing electron trapping within the HZO film. As a result, a wide memory window of 2.8 V was achieved.

As another approach to addressing performance degradation caused by the IL, research has also been conducted on FeFETs employing oxide semiconductors as the channel material [98–100]. By replacing the conventional Si channel with an oxide semiconductor and employing a low-temperature process (< 400 °C), the formation of the IL, which is typically produced by the oxidation of Si channel, can be suppressed. This approach also allows faster operation, reduces the operating voltage, and improves endurance. Kim et al*.* demonstrated a FeFET with an HZO ferroelectric layer and an indium zinc oxide (InZnO_x_) channel, integrating it into a 4 × 4 FeNAND array, as shown in the left panel of Fig. 5g [99]. The middle panel of Fig. 5g shows the cross-sectional structure of the device and the polarization states during write and inhibit operations. They presented a program/erase/inhibit scheme in a junctionless transistor–based FeNAND string utilizing the oxide semiconductor channel. The right panel of Fig. 5g presents the *I*_d_–*V*_g_ transfer curves for the program and erase states of all 16 cells in the integrated array. Furthermore, they demonstrated the scalability of this approach to 3D vertical channel structures, highlighting its potential for realizing highly integrated 3D FeNAND devices that suppress IL formation with low-temperature processing while achieving excellent device performance.

### Ferroelectric memcapacitor (FeCAP)

In addition to the three ferroelectric-based memory devices discussed above, the FeCAP, a two-terminal capacitor-based device utilizing ferroelectric memory, has recently attracted attention as an in-memory computing solution. A memcapacitor is a capacitor-based device with memory functionality implemented using non-volatile memory [101–106]. The switching operation of the memory induces a capacitance difference between memory states at the same voltage. A state exhibiting higher capacitance corresponds to the high-capacitance state (HCS), whereas a state with lower capacitance corresponds to the low-capacitance state (LCS). The ratio between the two capacitances at the read voltage (*C*_HCS_/*C*_LCS_) is referred to as the capacitance on/off ratio, while their difference (*C*_HCS_ − *C*_LCS_) is defined as the capacitive memory window (CMW). Unlike resistive devices, a memcapacitor performs vector–matrix multiplication (VMM) operations by summing the charges induced by its capacitance. The device is read by applying a read pulse, inducing a displacement current through the charging/discharging operation according to the relation *I* = *C* × d*V*dt ​, and integrating the induced current to obtain the total charge within the applied charging/discharging voltage range. Since a larger device capacitance induces more charge for the same read voltage range, a larger capacitive memory window between the HCS and LCS allows easier distinction between the two states. Therefore, widening the capacitive memory window is a critical performance metric for this kind of device. A memcapacitor array, implemented through a crossbar architecture of two-terminal capacitor devices, can achieve high integration density. Also, as capacitor devices consume only dynamic power, they exhibit negligible static power consumption. Furthermore, their relatively high intrinsic resistance compared with metal interconnects allows them to overcome limitations of resistive devices such as IR drop and sneak-path currents without the need for a select transistor [107, 108].

**Fig. 6:**
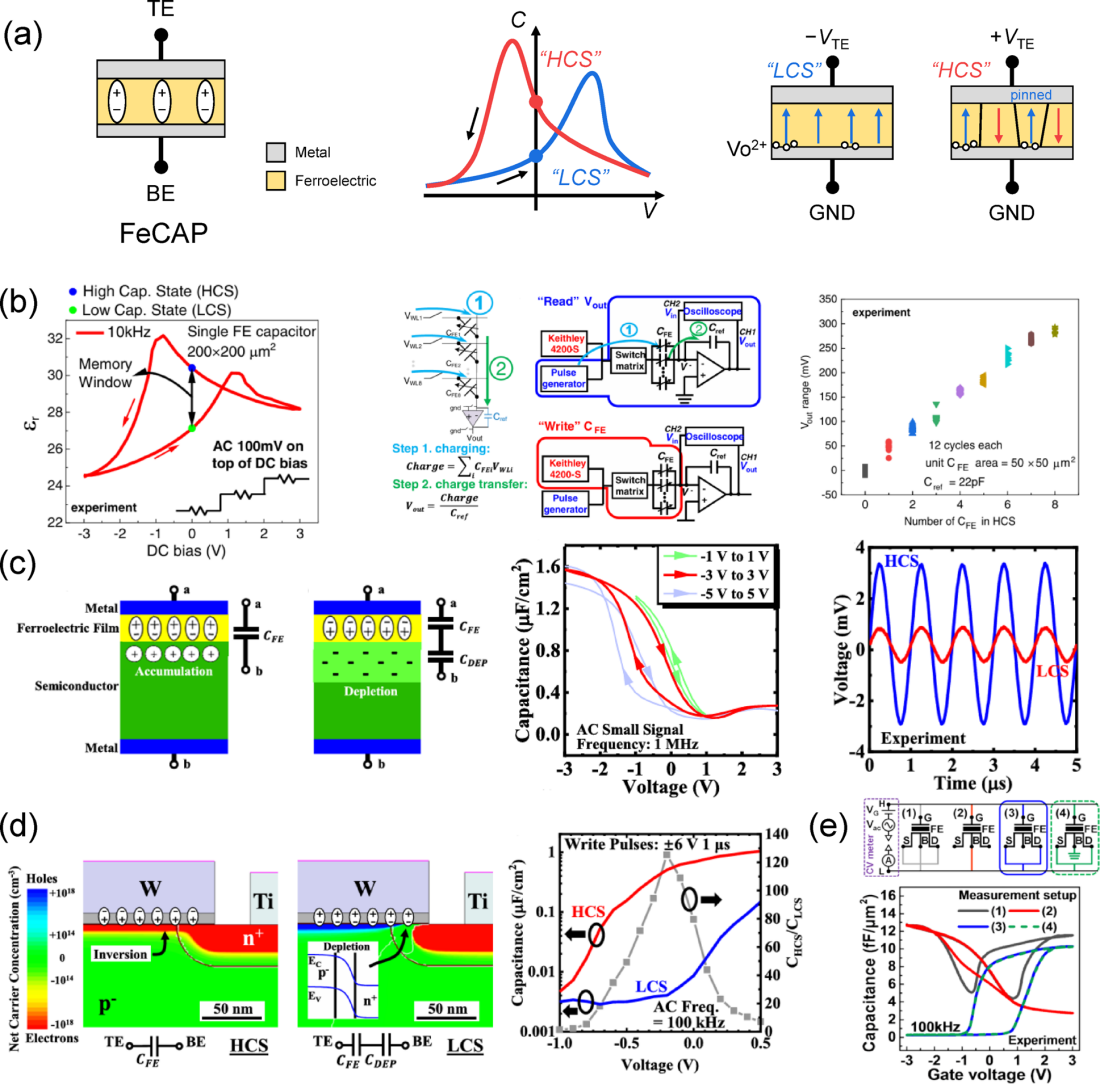
FeCAP. **a** Schematics of MFM FeCAP with *C*–*V* characteristics and the mechanism of asymmetric capacitance modulation. **b** Asymmetric *C*–*V* characteristics of an MFM FeCAP (left panel), charge-based VMM operation in a FeCAP crossbar array (middle panel), and VMM output voltage as a function of the number of high-capacitance states read (right panel). Reproduced under the terms of the CC-BY Creative Commons Attribution 4.0 International License from Ref [108]. Copyright 2022, John Wiley & Sons. **c** Operating principle of an accumulation-type MFS FeCAP (left panel), *C*–*V* characteristics obtained from a DC sweep (middle panel), and readout circuit output voltage for two capacitance states (right panel). Reproduced with permission from Ref [113]. Copyright 2020, IEEE. **d** Operating principle of an inversion-type MFS FeCAP (left panel) and *C*–*V* characteristics with capacitance ratio from a DC sweep (middle panel). Reproduced with permission from Ref [116]. Copyright 2023, IEEE. **e** Four capacitance measurement methods using FeFET devices (upper panel) and the corresponding *C*–*V* characteristics (bottom panel). Reproduced with permission from Ref [117]. Copyright 2023, IEEE

FeCAPs implement non-volatile memory functionality through the polarization of the ferroelectric layer and can be classified, based on their structural configuration, into two main types: MFM and MFS structures. The structure and operating principle of an MFM-based FeCAP are illustrated in Fig. 6a. In such MFM FeCAPs, excessive oxygen vacancies at the BE interface can induce domain wall pinning during the polarization switching process, leading to asymmetric small-signal capacitance–voltage (*C*–*V)* characteristics at DC 0 V [107, 109]. Alternatively, asymmetric *C*–*V* characteristics can be implemented by employing two metals with different work functions as the TE and BE, thereby utilizing interfacial asymmetry [110, 111]. The device exploits the capacitance difference observed at DC 0 V to define the memory window between the two states, utilizing the difference in induced charge from small-signal or read-pulse within this voltage range to perform VMM operations. Since this readout method does not change the device state, the structure is identical to that of FeRAM, but it differs in that the device state is sensed by a nondestructive read operation. Hur et al. demonstrated a TiN/HZO/TiN MFM FeCAP and a 12 × 12 crossbar array [107]. The *C*–*V* characteristics of the device, shown in the left panel of Fig. 6b, reveal an on/off ratio of 1.125 at DC 0 V under 10 kHz, 100 mV AC small-signal readout, resulting from the asymmetric curve. The middle panel of Fig. 6b illustrates the charge-based VMM operation in a FeCAP crossbar array. As described earlier, charging and discharging read operations are performed through the selected WLs of the crossbar, and the charges generated in each device are summed along the BLs. These summed charges are then converted into an output voltage through an op-amp based charge integrator circuit. The right panel of Fig. 6b presents the VMM operation results measured in the 12 × 12 array, showing a linear increase in the output signal with the number of HCS devices engaged in the read operation.

An MFS-based FeCAP operates in a manner similar to utilizing the ferroelectric gate stack capacitance (*C*_FE_) of a FeFET, with its operating mechanism varying depending on the positions of the two electrodes. In the accumulation-type MFS FeCAP, the basic principle is derived from the *C*–*V* characteristics of a MOS capacitor, where the polarization of the ferroelectric layer shifts the flatband voltage, thereby inducing either depletion or accumulation in the underlying Si body at the same applied voltage [112, 113]. When the device is in the accumulation region, only the *C*_FE_ is observed, corresponding to the HCS. Conversely, when the device switches to the depletion state, the series connection of the Si depletion capacitance (*C*_DEP_) and the *C*_FE_ results in the LCS. Zhou et al. demonstrated an accumulation-type FeCAP using an W/HfAlOx/p-Si MFS stack [112]. The left panel of Fig. 6c illustrates the principle of capacitance modulation based on the polarization of the ferroelectric layer in an accumulation-type FeCAP. The middle panel in Fig. 6c shows the *C*–*V* characteristics over a range of DC sweep voltages, where both the typical MOS capacitor *C*–*V* profile and the flatband voltage shift induced by ferroelectric polarization, along with the resulting memory window, can be observed. Furthermore, through a DC sweep measurement from – 3 to 3 V under a 1 MHz AC small signal, an on/off ratio of ~ 5 was demonstrated at 0 V. The right panel in Fig. 6c presents the transient response measured using a resistor-based readout circuit with a 100 mV, 1 MHz small-signal sine wave. The results confirm that the difference in capacitance between the two states leads to a corresponding difference in the output voltage.

The second operating mechanism of the MFS-based FeCAP, referred to as the inversion-type FeCAP, operates by configuring the gate electrode and the heavily doped region surrounding the cell as the BE [114–117]. Unlike the accumulation-type FeCAP, which employs a lightly doped Si body as the BE, this structure utilizes an ion implantation process to form a heavily doped region around the cell. This region slightly overlaps with the device area, enabling a variation in the observed capacitance depending on the switching state of the ferroelectric layer. Zhou et al. demonstrated an inversion-type FeCAP based on a W/HfAlO_x_/p-Si MFS stack, where an n⁺-implanted region was formed around the p-Si body, and further experimentally validated a 32 × 32 crossbar array, as shown in the left panel of Fig. 6d [114, 115]. When a positive write voltage is applied to the device, the channel undergoes inversion, resulting in the HCS characterized by the *C*_FE_. Conversely, when a negative voltage is applied, the channel enters depletion, and the series connection between the depletion capacitance of the overlapped depletion region *C*_DEP_ and *C*_FE_ produces the LCS. The *C*_LCS_ in this case is significantly smaller than that of the accumulation-type FeCAP, which utilizes the *C*_DEP_ of the entire cell area, achieving a large *C*_HCS_/*C*_LCS_ on/off ratio. The right panel of Fig. 6d shows the DC *C*–*V* curves of each state, written with ± 6 V, 1 μs pulses and measured with a 100 kHz AC small signal, along with the on/off ratio as a function of the read voltage. A maximum on/off ratio of ~ 125 is observed near 0 V. Furthermore, this inversion-type FeCAP provides carriers for both switching directions, enabling switching operation in the nanosecond range. Under a positive write pulse, minority carrier electrons are supplied to the surface from the n⁺-doped region, whereas under a negative write pulse, holes are injected via band-to-band tunneling and trap-assisted tunneling induced by strong band bending in the overlapped region. This inversion-type FeCAP can also be implemented by utilizing the FeFET gate stack capacitance in conjunction with the n⁺-heavily doped source/drain regions as electrodes [116, 117]. Kim et al. demonstrated FeCAP operation using GlobalFoundries 28 nm node n-type FeFET [116]. Figure 6e illustrates four terminal connection configurations for the FeFET, and presents the corresponding *C*–*V* measurement results. When the source/drain region was used as the BE, the device exhibited the same operating principle as the inversion-type FeCAP, where the LCS was determined solely by the capacitance of the source/drain overlap region. This configuration achieved an on/off ratio of ~ 25 at DC 0 V under a 100 kHz AC small signal measurement, demonstrating both the feasibility and the scalability of implementing FeCAP devices using FeFET technology.

## In-Memory computing with ferroelectric devices

### Neuromorphic computing

The limitations of the conventional von Neumann architecture, such as the memory processing bottleneck and high energy consumption, have motivated the exploration of alternative computing paradigms. Neuromorphic computing, which emulates the architecture and operating principles of the human brain, offers efficient, parallel, and adaptive information processing [118–122]. A key requirement for hardware-based neuromorphic computing is the development of artificial synaptic devices capable of reliably emulating the weight modulation of biological synapses [123–127]. In hardware neural networks, the weight adjustment of artificial synapses is typically achieved by modulating the conductance of memory devices. Therefore, the potentiation and depression processes, which gradually increase or decrease the device conductance to match synaptic weight updates, are critical. Ideally, these conductance changes should be linear and symmetric, with minimal variations arising from hardware non-idealities [128]. Moreover, achieving precise weight representation and fine conductance modulation requires a multi-level capability in the device, enabling higher accuracy in neural network computations. For stable operation, artificial synaptic devices must also exhibit high endurance for repeated conductance updates and robust nonvolatile retention characteristics to maintain weights over extended periods. Therefore, extensive research efforts have explored neuromorphic computing implementations using a wide range of emerging nonvolatile devices, each offering distinct switching mechanisms, energy efficiencies, and scalability advantages [129–138].

Ferroelectric-based devices maintain spontaneous polarization for extended periods without the need for an external voltage, offering nonvolatile characteristics with lower power consumption and faster switching speed compared to conventional charge trap flash memories. These advantages make them well suited for hardware neural network implementations that require low power, large-scale integration, and high-speed computation. By tuning the amplitude or width of the write pulse, the portion of aligned polarization can be finely controlled, enabling multi-state operation and allowing precise weight modulation in artificial neural networks [139]. In particular, HfO_2_-based ferroelectric devices exhibit high compatibility with standard CMOS fabrication processes and excellent scalability, providing strong potential for the commercialization of highly integrated arrays. In this section, recent studies on hardware implementations of artificial synapses using hafnia-based ferroelectric memory devices are discussed.

**Fig. 7:**
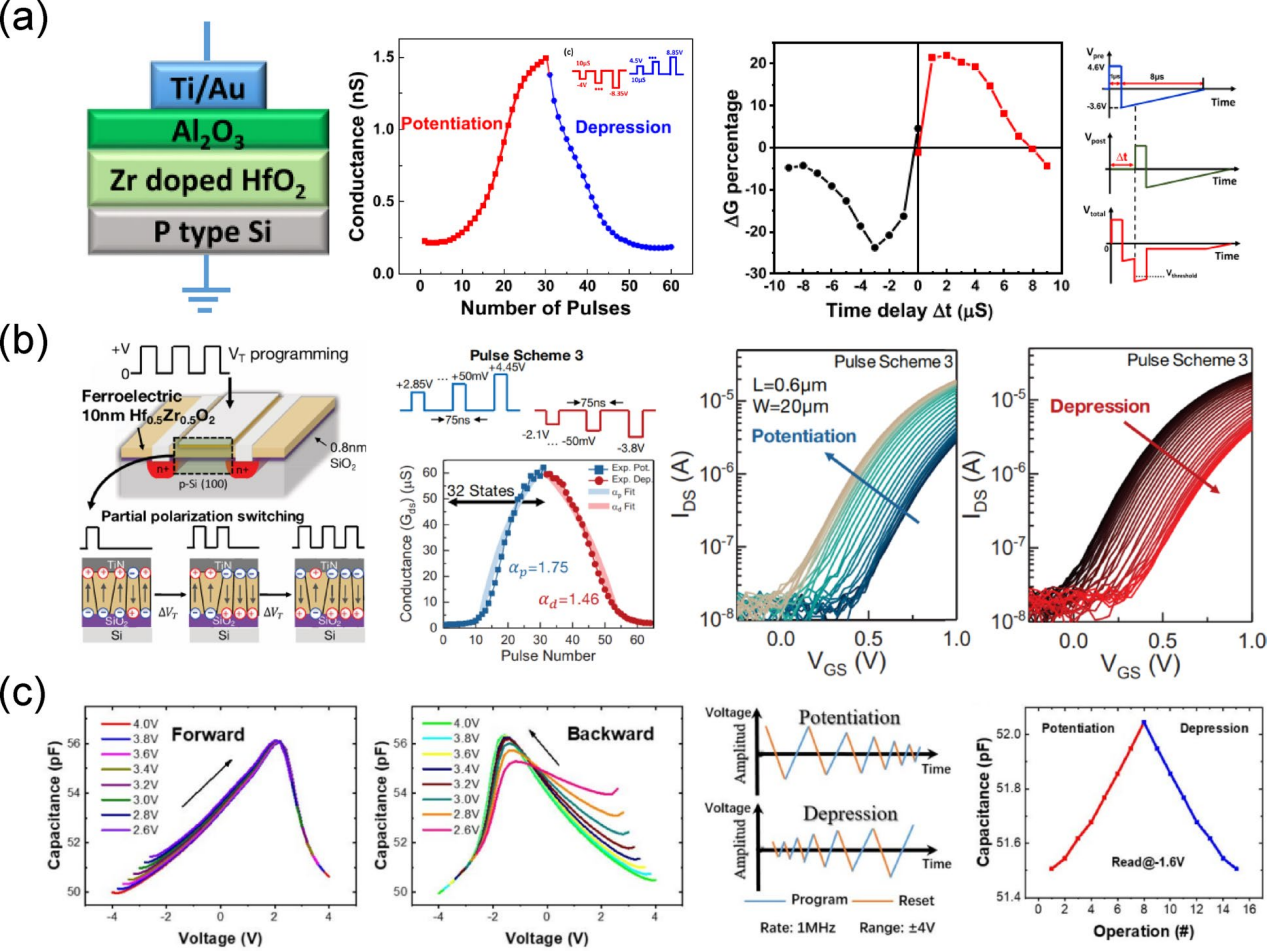
**a** Schematic structure and synaptic behavior of a FTJ based on an Al_2_O_3_/Zr-doped HfO_2_ stack for neuromorphic computing. (Left panel) Cross-sectional schematic showing the Ti/Au TE Al_2_O_3_ IL, Zr:HfO_2_ ferroelectric layer, and p-type Si substrate. (Middle panel) Potentiation and depression characteristics under repeated pulse stimulation, demonstrating conductance modulation. (Right panel) STDP response as a function of time delay (Δt) between pre- and post-synaptic spikes, along with pulse schemes for potentiation and depression. Reproduced with permission from Ref [141]. Copyright 2019, Springer Nature. **b** FeFET analog synapse for accelerating deep neural network training. (Left panel) Device schematic and mechanism of partial polarization switching in a FeFET with a 10 nm Hf_0.5_Zr_0.5_O_2_ ferroelectric layer and 0.8 nm SiO_2_ IL. (Middle panel) Multilevel conductance modulation (32 states) obtained using a specific pulse scheme in which the gate voltage is incrementally stepped by ± 50 mV per pulse. (Right panel) Transfer characteristics (*I*_DS_–*V*_GS_) showing gradual changes during potentiation and depression cycles. Reproduced with permission from Ref [153]. Copyright 2017, IEEE. **c** (Left panel) *C*–*V* curves in both forward and backward sweeps under various programming voltages. (Middle panel) Schemes of the program and reset voltage waveforms for potentiation and depression, operated at a rate of 1 MHz and within a ± 4 V range. (Right panel) Capacitance modulation during repeated potentiation and depression cycles, measured at a read voltage of – 1.6 V. Reproduced with permission from Ref [163]. Copyright 2019, IEEE

First, as discussed above, FTJs are two-terminal devices in which a thin ferroelectric layer serves as the tunneling barrier between two electrodes. Their tunneling conductance can be modulated by reversing the ferroelectric polarization, enabling non-volatile, analog-like resistance states. Specifically, potentiation is achieved by pulses that switch the polarization to lower the tunneling barrier, which increases the device conductance and drives it toward the LRS. Conversely, depression is induced by pulses that switch the polarization to raise the tunneling barrier, which decreases the device conductance and drives it toward the HRS. These characteristics render FTJs highly attractive for neuromorphic computing, where gradual and multi-level conductance modulation is essential for emulating biological synaptic weights. [140–145]. Furthermore, the tunneling-based switching mechanism offers sub-nanosecond operation speeds and ultra-low read energy, reinforcing their potential for large-scale neuromorphic processors. Figure 7a illustrates a representative FTJ device structure, composed of Ti/Au as the TE, an Al_2_O_3_ IL, Zr-doped HfO_2_ as the ferroelectric layer, and p-type Si as the BE [140]. The inclusion of an ultrathin Al_2_O_3_ layer improves interface quality and helps achieve stable tunneling characteristics. The potentiation and depression characteristics were demonstrated by applying sequences of voltage pulses to gradually modulate the device conductance using three different schemes. The first scheme used constant amplitude and width, the second used constant amplitude while varying the pulse width, and the third used constant width while gradually increasing the pulse amplitude. The middle panel of Fig. 7a presents the conductance modulation obtained with the third scheme, where a pulse width of 10 μs was maintained. For potentiation, the amplitude was increased from – 4 to – 8.35 V, and for depression from 4.5 to 8.85 V. As a result, the device exhibits clear and symmetric potentiation and depression characteristics across multiple conductance states, which is critical for accurate weight updates in artificial neural networks. The right panel of Fig. 7a demonstrates spike-timing-dependent plasticity (STDP), where the conductance change (Δ*G*) is plotted against the relative timing difference (Δ*t*) between pre-synaptic and post-synaptic voltage spikes. Positive Δ*t* results in potentiation, while negative Δ*t* leads to depression. Such behavior enables unsupervised learning and associative memory functions in hardware-based neuromorphic systems, arising from the time-dependent polarization switching dynamics in the ferroelectric layer, where the overlap of voltage waveforms determines the net electric field and thus the extent of domain reorientation.

However, challenges remain in achieving linear and symmetric conductance updates over many cycles, mitigating device-to-device (D2D) variation, and ensuring long-term retention in ultra-thin ferroelectric layers. To overcome these limitations, recent research has pursued several engineering approaches. Interface engineering has been extensively explored to optimize both the ferroelectric and tunneling layers, enhancing endurance, retention, and ON/OFF ratios through careful control of interfacial quality and dielectric placement within the Hf_0.5_Zr_0.5_O_2_ stack [146, 147]. Such strategies not only stabilize the tunneling barrier but also improve the linearity and symmetry of conductance modulation, which is critical for reliable synaptic weight updates. In parallel, ferroelectric property recovery techniques such as thermal re-wake-up have been proposed to restore degraded polarization states, enabling the recovery of high ON/OFF ratios and stable operation in scaled FTJs [148]. From a fabrication perspective, CMOS-compatible process integration has been demonstrated to realize low-power FTJs suitable for dense neuromorphic synapse arrays without sacrificing device performance [80]. Additionally, structural and stress engineering approaches have emerged as effective tools to tailor crystallographic orientation, domain distribution, and imprint fields, resulting in improved self-rectification, reduced leakage currents, and enhanced multilevel switching behavior [149, 150]. These advancements indicate that FTJ-based synaptic devices are steadily advancing toward practical, large-scale neuromorphic hardware, integrating high integration density, low operating energy, and analog-like weight storage in a crossbar architecture.

FeFET implements potentiation and depression by applying a write voltage to the gate, which switches the ferroelectric polarization, thereby modulating the device *V*_th_ and channel conductance, and consequently controlling the drain current. Compared to two-terminal structures, the three-terminal configuration offers greater immunity to sneak path currents and IR drop, enabling more accurate VMM operations. Consequently, extensive research has focused on implementing neuromorphic systems using FeFET devices [151–160]. Jerry et al. demonstrated analog synaptic behavior using an MFIS FeFET with a 10 nm HZO layer [151]. As shown in the left panel of Fig. 7b, the channel conductance of the FeFET can be tuned by the gate voltage, and by optimizing pulse number, amplitude, interval, and width, partial polarization can be induced to achieve gradual programming. Three pulse schemes were examined, including applying identical pulses with fixed width and amplitude, applying pulses with fixed amplitude while increasing the width, and applying pulses with fixed width while increasing the amplitude. The middle panel of Fig. 7b presents the conductance modulation obtained using the third scheme. Both potentiation and depression were performed with a constant pulse width of 75 ns, where the amplitude was increased from 2.85 to 4.45 V for potentiation and from – 2.1 to – 3.8 V for depression. This scheme exhibited linear modulation across 32 distinct conductance states as a function of the number of applied pulses. In addition, the non-linearity (α) of the potentiation and depression curves was analyzed as a function of the pulse number. While an ideal α is 0, the extracted non-linearity values were 1.75 for potentiation and 1.46 for depression. The right panel of Fig. 7b presents the *I*_d_–*V*_g_ transfer curves corresponding to the potentiation and depression processes. Through careful optimization of pulse conditions, the device achieved 32 conductance states with excellent symmetry and linearity, resulting in an MNIST online learning simulation accuracy of 90%. Mulaosmanovic et al. demonstrated a synaptic device using a TiN/Si:HfO_2_/SiON/Si gate stack MFIS FeFET fabricated with 28 nm HKMG technology and a 500 nm channel length [152]. Gradual channel conductance modulation was achieved through both amplitude and width modulation of the gate pulses. The single FeFET structure incorporated hafnium oxide as the active material in series with a resistor. By tuning the resistance value and the spiking duration, the device exhibited both spike transmission and synaptic plasticity through STDP. Sun et al. presented a hybrid in-situ training and inference scheme implemented with a 2 T–1FeFET weight unit cell using an HZO FeFET [153]. In this approach, during training the volatile gate voltage of the FeFET was used to represent the least significant bit, while during inference the nonvolatile polarization state stored information corresponding to the most significant bit. Their device exhibited multi-level conductance with 64 states, high symmetry, and linearity in conductance updates, achieving 97.3% recognition accuracy on the MNIST dataset. Seo et al. demonstrated a junctionless-type FeFET synaptic transistor with a FinFET structure incorporating an 8.5 nm HZO layer [154]. By applying identical pulses, the device achieved 32 distinct conductance states, enabling an MNIST recognition accuracy of 80%. Although the recognition rate was lower compared to other works due to the use of a non-optimized pulse scheme, this study highlighted the significance of applying a cost-effective, logic-process-compatible synaptic device based on a junctionless FinFET structure for neuromorphic applications.

FeCAP-based synaptic devices can utilize the high integration density of the 4F^2^ crossbar array architecture while suppressing non-idealities such as IR drop and sneak path currents thanks to the nature of a capacitor, thereby minimizing computational errors. In addition, they consume negligible static power, offering significant advantages for large-scale synaptic operations and demonstrating strong potential for neuromorphic computing applications [107, 108]. Zheng et al. demonstrated the TiN/Al:HfO_2_/TiN (MFM) FeCAP device as a synaptic device and reported a capacitive neural network based on a FeCAP crossbar array [161]. The left panel of Fig. 7c shows the *C*–*V* characteristics for the two polarization states of the MFM FeCAP under forward and backward sweeps, as well as its multilevel capacitance behavior.

The middle panel of Fig. 7c shows the program and reset voltage waveforms used to induce potentiation and depression, distinct from the pulse schemes typically adopted in conductance-based devices. Before adjusting the capacitance state, both potentiation and depression start with a reset step that initializes the polarization to + *P*_r_ by applying a backward sweep with an amplitude larger than the coercive field. Potentiation is then achieved by a subsequent set step in which the polarization is reversed from + *P*_r_ to – *P*_r_ through a reduced negative program voltage in the forward direction, resulting in a gradual increase in capacitance. Depression, on the other hand, is induced by applying program voltages with progressively larger amplitudes, which further enhance the polarization and thereby decrease the capacitance. After each programming sequence, capacitance modulation is measured at – 1.6 V in forward sweep mode. The right panel of Fig. 7c presents the readout results for potentiation and depression performed under a ± 4 V range at 1 MHz, demonstrating symmetric and nearly linear modulation across multiple states. Building on this, the FeCAP crossbar array was integrated with an op-amp-based charge integrator circuit, enabling charge-based VMM operations within the synapse array and voltage output generation in the neuron circuit. With 3-bit weight resolution, the proposed system achieved an MNIST recognition accuracy of 78.28%, demonstrating the feasibility of implementing capacitive device-based artificial neural networks using FeCAP devices and arrays.

### Hardware-based security

The rapid advancement of IoT and cloud computing technologies has led to widespread interconnection among edge devices, enabling the exchange of vast amounts of data across networks [162–164]. While such interconnectivity has driven automation and substantial efficiency gains across diverse industries, it has also exposed sensitive information, including personal data in healthcare and finance, to heightened hacking risks, thereby increasing the demand for more robust and reliable security and encryption technologies beyond conventional software-based approaches [165, 166]. Recently, research efforts have increasingly focused on exploiting the intrinsic entropy of hardware for encryption and random number generation. Representative hardware-based security primitives include the true random number generator (TRNG), which utilizes hardware entropy to produce random numbers, cryptographic keys, and ciphers [167], and the PUF, which exploits inherent process variations from semiconductor fabrication process to generate unique device fingerprints and corresponding challenge–response pairs (CRPs) [168, 169]. As edge devices continue to scale down in size, conventional hardware security solutions that rely on large-area, complex, and high-power circuitry face intrinsic limitations. This scaling trend has increased the demand for compact, energy-efficient hardware security architectures, thereby stimulating growing interest in various NVM-based approaches. [167, 170–181]. Ferroelectric memory-based devices, with their non-volatile characteristics that enable long-term data retention without external power, low-power operation, excellent compatibility with existing CMOS processes, and proven scalability, present strong potential for next-generation hardware security systems.

**Fig. 8:**
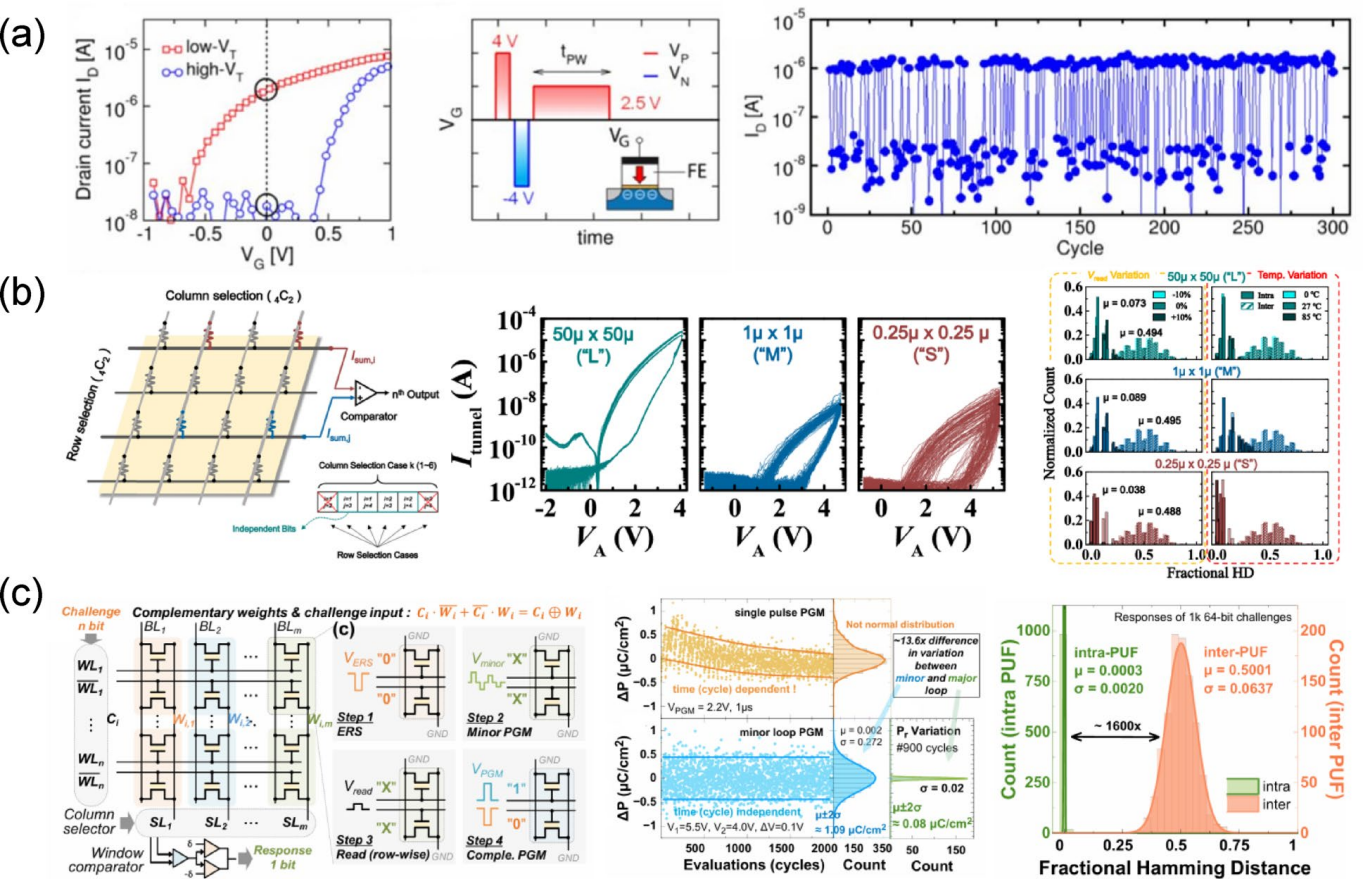
**a** *I*_d_–*V*_g_ transfer curve of a FeFET (left panel), gate-voltage pulse scheme used to exploit C2C variation in switching pulse time (middle panel), and drain current read at *V*_g_ = 0 V after 300 repeated random program operations (right panel). Reproduced with permission from Ref [184]. Copyright 2018, IEEE. **b** Schematic of a PUF design using a 4 × 4 FTJ crossbar array and cryptographic code generation method (left panel), *I*–*V* characteristics of FTJ devices with three different cell sizes and D2D variation across 100 devices (middle panel), and inter- and intra-HD of PUFs implemented with three different FTJ sizes with various read voltages and temperature (right panel). Reproduced with permission from Ref [185]. Copyright 2021, IEEE. **c** PUF design using a 2 T-FeFET and its enrollment and reconfiguration processes (left panel), comparison of mid-polarization state variation obtained via single-pulse and minor-loop programming (middle panel), and intra- and inter-HD of PUFs implemented using minor-loop programming in 2 T-FeFETs (right panel). Reproduced with permission from Ref [187]. Copyright 2023, IEEE

Mulaosmanovic et al. demonstrated a random number generator by exploiting the cycle-to-cycle (C2C) switching variation of a poly-Si/TiN/HfO_2_/SiON gate stack FeFET fabricated using a 28 nm HKMG process [182]. The left panel of Fig. 8a shows the transfer characteristics of a 30 nm gate length device in both program and erase states, exhibiting an on/off ratio exceeding 100 at 0 V. The middle panel of Fig. 8a illustrates the gate voltage pulse sequence used to extract switching variations, where the device was first set to a reference high *V*_th_ state by sequentially applying a 4 V program pulse and a − 4 V erase pulse, followed by application of a 2.5 V program pulse. The time required for the device to switch from the high *V*_th_ state to the low *V*_th_ state was then recorded. When the channel length is scaled down to dimensions comparable to the ferroelectric domain size, the gate stack contains only one or a few switching domains. Under these conditions, a small number of dominant polarization switching events can abruptly change the device state during voltage application. To capture this behavior, 50 consecutive C2C measurements were performed, revealing that approximately 50% of switching events occurred at pulse widths near 10 μs. The right panel of Fig. 8a presents the results obtained by applying a 2.5 V pulse with a 9.1 μs width over 300 consecutive cycles, showing the device state and drain current for each cycle. Due to the 50% switching probability, the device generated random sequences of switching and non-switching events, thereby demonstrating that the inherently stochastic polarization switching of ferroelectric domains can be harnessed to generate unclonable bit sequences.

FTJ-based PUFs utilize intrinsic tunneling current variability arising from D2D differences in ferroelectric polarization states and barrier profiles to produce unique and reproducible digital fingerprints. The left panel of Fig. 8b shows a simulated 4 × 4 crossbar array of TiN/Hf_0.5_Zr_0.5_O_2_/TiN FTJs, where column and row selection lines route read currents to a comparator for bit generation (Kim et al. [183]). In the middle panel of Fig. 8b, D2D variability is characterized by tunneling current measurements at a fixed read voltage for devices with lateral dimensions of 50 × 50, 1 × 1, and 0.25 × 0.25 µm^2^. Here, smaller devices exhibit greater relative variation due to stronger local inhomogeneity effects, enhancing uniqueness. The right panel of Fig. 8b presents fractional Hamming distance (HD) distributions, showing inter-device HD values near the ideal 0.5, indicating statistical independence of responses, and intra-device HD values between 0.038 and 0.089 under ± 10% read-voltage variation and temperature changes from 0 to 85 °C, confirming stable performance. These results highlight the potential of FTJ-based PUFs to deliver high entropy, strong repeatability, and CMOS compatibility for secure, low-power, and AI-integrated hardware systems. Yu et al*.* demonstrated FTJ-based PUFs using crossbar arrays with device areas of 0.6, 1.2, and 2.0 µm^2^ to study the effect of scaling on performance [184]. Smaller devices showed broader current distributions from stronger local defects and domain variation effects, enhancing uniqueness. Inter-HD values remained near 0.5 for all sizes, while intra-HD stayed low, confirming high reliability. This highlights that scaling can balance uniqueness and stability without added circuitry, supporting the scalability of FTJ-based PUFs for secure, low-power authentication.

**Fig. 9:**
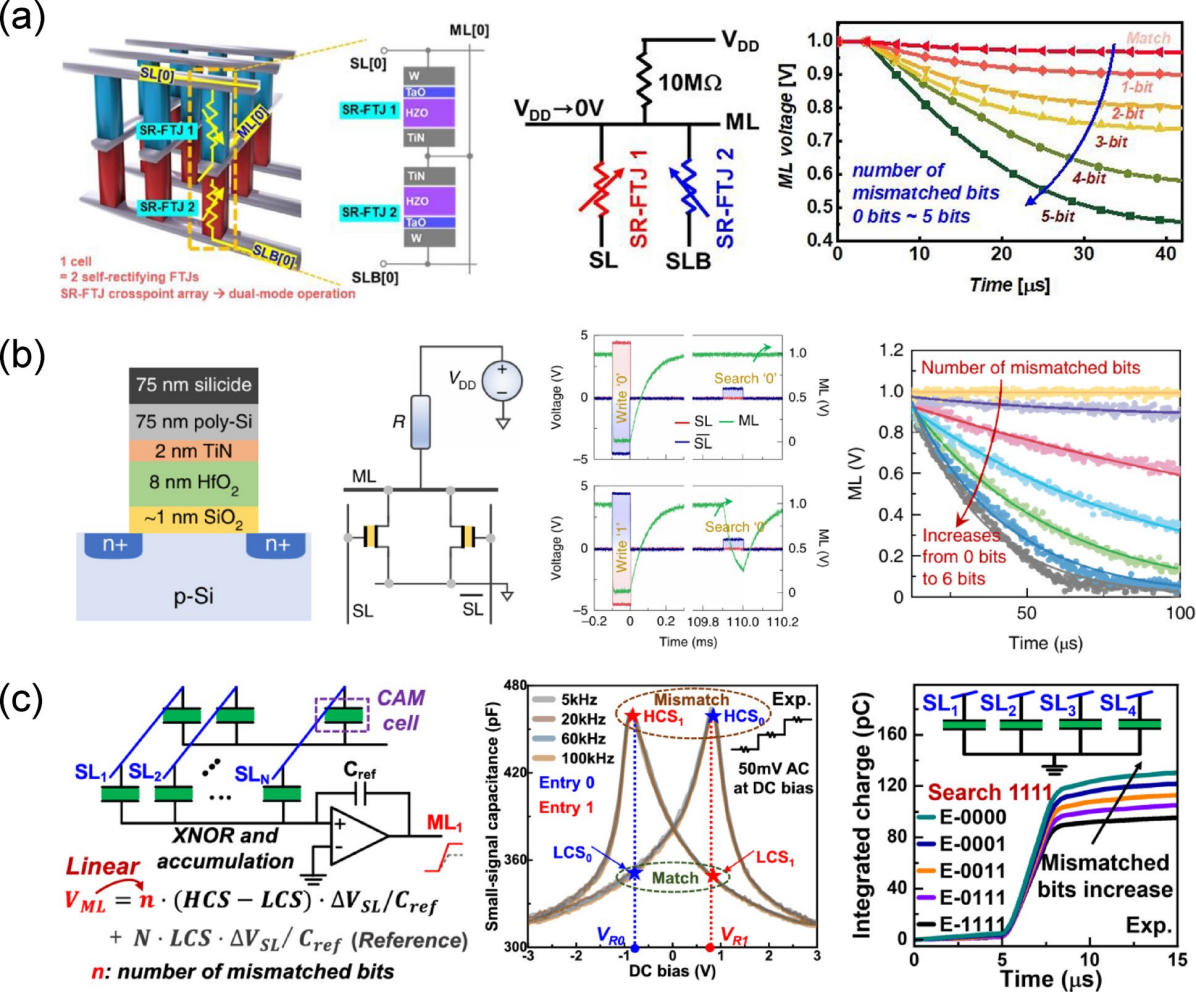
**a** Schematic of a cross-point array composed of two self-rectifying FTJ cells (left panel), TCAM cell structure based on two self-rectifying FTJs (middle panel), and ML voltage results as a function of the number of mismatch bits (right panel). Reproduced with permission from Ref [208]. Copyright 2023, IEEE. **b** Cross-sectional view of a FeFET device and schematic of a 2-FeFET TCAM single cell (left panel), ML voltage results during the search operation of a single TCAM cell (middle panel), and ML voltage discharge characteristics as a function of mismatch bit count in a 6-bit TCAM array (right panel). Reproduced with permission from Ref [194]. Copyright 2019, Springer Nature. **c** CAM structure using a FeCAP crossbar array (left panel), small-signal *C*–*V* characteristics of an MFM FeCAP device along with device states and search scheme (middle panel), and integrated charge results as a function of mismatch bit count in a 1 × 4 CAM array (right panel). Reproduced with permission from Ref [218]. Copyright 2024, IEEE

Shao et al. demonstrated a PUF using a 10 × 10 AND-type FeFET array with a HAO/Al_2_O_3_ FE/IL stack, leveraging ferroelectric polarization variation induced by minor-loop program as the entropy source [185]. The PUF design and CRP generation scheme are illustrated in Fig. 8c (left panel). In the array, each column contains a cell consisting of two FeFETs, which are programmed into complementary low-*V*_th_ and high-*V*_th_ states, while complementary high and low gate voltages are applied through two WLs. In this configuration, current flows only when a low-*V*_th_ state is driven by a high gate voltage, effectively implementing an XOR operation. The XOR operations of individual two-FeFET cells are applied simultaneously to *n* complementary WLs, resulting in 2^*n*^ possible cases. The currents from individual cells are summed along with the SLs, representing both the aggregate XOR output and the total on-current. From *m* independent SLs, _*m*_C_2_ combinations of current comparisons are performed, and a window-comparator-based scheme is used to generate the final 1-bit response. The entropy source for each cell is established by programming it into a random mid-polarization state, thereby exploiting random D2D polarization variations. This mid-polarization state is achieved through a minor-loop programming in which program and erase voltages are applied sequentially with gradually decreasing amplitudes, leading to a final random polarization configuration via domain pinning. The middle panel of Fig. 8c compares the variations obtained from 2000 repeated program operations using a single pulse versus minor-loop programming. Devices programmed via the minor-loop method exhibited approximately 13.6 × greater variation. Furthermore, the high uniformity achieved through major-loop programming ensured a stable and sustainable entropy source, resulting in superior reconfigurability. The right panel of Fig. 9c presents the intra- and inter- HD distributions of the CRPs generated by the proposed PUF system, showing excellent reproducibility and uniqueness. These results highlight that the combination of high variation from ferroelectric-domain minor-loop programming and the nonlinearity introduced by XOR operations enables the realization of a highly random and robust PUF architecture. Li et al. demonstrated a reconfigurable PUF using a doped HfO_2_-based 2FeFET-1C cell array fabricated in a 28 nm technology node, exploiting C2C variation as the entropy source [186]. Through charge-domain computation enabled by the capacitor in the 2FeFET–1C cell, the design achieved an energy efficiency of 1.89 fJ per bit, significantly lower than that of other state-of-the-art strong PUFs, while also delivering excellent area efficiency of 1 μm^2^ per cell owing to the 28 nm technology and compact cell architecture.

### Associative memory

Another in-memory computing paradigm that has attracted significant attention is associative memory, also known as content-addressable memory (CAM). This architecture enables massively parallel search operations across an entire memory array in a single search operation, allowing direct data retrieval based on content rather than specific addresses [187]. Such capability makes CAM particularly suitable for data-intensive tasks, offering high-speed and energy-efficient search operations that can significantly reduce the latency and energy overhead associated with data movement in conventional computing systems. CAM architectures can be categorized into binary content-addressable memory (BCAM), which stores and searches binary data, and ternary content-addressable memory (TCAM), which incorporates an additional “X” (don’t care) bit to provide more flexibility in search operations. Traditionally, CAM has been employed in various applications such as network routing and packet classification [188–191]. More recently, its search capability has been utilized in emerging machine learning domains, including memory-augmented neural networks (MANNs), and used as a HD computing kernels [192–194]. Conventional CAM and TCAM implementations are typically based on SRAM, where a single TCAM cell consists of 16 transistors [195, 196]. However, with the growing demand for storing and searching increasingly large datasets, conventional SRAM-based CAM faces inherent limitations arising from its large cell area and high energy consumption. To address these challenges, recent studies have explored NVM-based TCAM architectures that offer compact cell designs, low power consumption, and high-speed search capabilities [197–205]. Among these, ferroelectric memory has gained attention due to its low-power operation, high-speed, CMOS compatibility, and suitability for large-scale integration using commercially scalable technologies. In this section, we review recent advances in ferroelectric memory-based CAM research.

FTJ-based CAM combines nonvolatility with low-power operation, supporting energy-efficient, high-speed parallel search [83, 206, 207]. As shown in Fig. 9a, Lim et al. presented an associative memory architecture based on a self-rectifying ferroelectric tunnel junction (SR-FTJ) crosspoint array, where the Hf_0.5_Zr_0.5_O_2_ ferroelectric layer serves as the tunneling barrier and asymmetric interfaces induce built-in rectification to suppress sneak-path currents, eliminating the need for additional selector devices [206]. The circuit schematic depicts each cell integrating two SR-FTJs connected in opposite polarities for dual-mode operation, with a load resistor on the match-line (ML) to sense current differences, enabling direct comparison between input and stored patterns. The measured ML voltage transients reveal that the voltage decay rate is strongly dependent on the number of mismatched bits, with perfect matches exhibiting minimal voltage drop and patterns with greater mismatches showing faster decay. During associative search operations, the applied input pattern produces BL currents that sum according to Ohm’s and Kirchhoff’s laws, and the stored pattern yielding the smallest voltage drop is identified as the correct match. The demonstration of CAM operations using a 32 × 32 SR-FTJ array successfully matched binary patterns without significant leakage, confirming the suitability of SR-FTJ arrays for high-density, nonvolatile, and selector-free associative memory in AI and IoT applications.

FeFET-based TCAM has also emerged as a promising solution that combines the benefits of non-volatile storage, fast switching speed, and low power operation, enabling compact cell design and high-density integration using CMOS compatible fabrication technology. Utilizing these advantages over conventional flash devices, recent research has demonstrated highly scalable TCAM implementations using FeFETs [192, 193, 208–215]. Ni et al. demonstrated a 2FeFET TCAM using an industrial 28 nm HKMG FeFET process [192]. The left panel of Fig. 9b shows the cross-sectional view of the fabricated FeFET device with an 8 nm doped HfO_2_ ferroelectric layer and the schematic of a single TCAM cell. In this design, two FeFETs are programmed into complementary *V*_th_ states, with one device in a high *V*_th_ state and the other in a low *V*_th_ state. The search operation is carried out by applying complementary voltages, the read voltage and 0 V, to the two gates associated with the search lines (SL and SL′) and prior to the search, the ML is precharged. In the match case, the read voltage is applied to the high *V*_th_ device, which remains in cut-off and prevents discharge, thereby maintaining the precharged ML voltage. In contrast, in the mismatch case, the read voltage is applied to the low *V*_th_ device, which turns on and discharges the ML to ground. The right panel of Fig. 9b presents search operation results for a 1 × 6 TCAM array, showing that the ML discharges more rapidly as the number of mismatch bits increases. Ni et al. further investigated the linear relationship between ML discharge rate according to the mismatch bit, exploiting this HD computation capability for implementing a MANN, which was demonstrated on the Omniglot dataset. Benefiting from the compact 2FeFET cell design and a commercial technology node, their TCAM achieved an ultra small cell area of 0.15 μm^2^, along with low search delay and search energy, demonstrating an area and energy efficient TCAM architecture.

Lastly, research efforts have also explored implementing parallel CAM search operations using FeCAP crossbar arrays in a charge-based approach [216–218]. Xu et al. demonstrated a 1FeCAP CAM cell utilizing a TiN/HZO/TiN MFM FeCAP, with the array configuration and operating principle schematically illustrated in the left panel of Fig. 9c [216]. The *C*–*V* characteristics of the device, shown in the middle panel of Fig. 9c, reveal that for the two binary states, the device exhibits distinct HCS and LCS regions under two specific search voltage levels, from which the stored value is read using a small-signal measurement. In this scheme, a stored bit matching the search bit results in the device being in the LCS, producing a low readout charge, whereas a mismatch sets the device to the HCS, yielding a higher charge. By applying small-signal search voltages corresponding to the search data to the TEs (SLs), the resulting charges from all devices flow individually to the BEs, where they are accumulated by an op-amp integrator for sensing. Consequently, the total accumulated charge increases linearly with the number of mismatched bits, as experimentally demonstrated in a 1 × 4 CAM array shown in the right panel of Fig. 9c. Leveraging this linear HD computation, Xu et al. further implemented a MANN. Owing to the 4F^2^ footprint, FeCAP-based CAM enables high-density array integration, while offering low-power and high-reliability charge-based computation. Finally, when scaled to a 45 nm technology node, the design was estimated to achieve a search energy only 0.006 times that of conventional SRAM-based CAM.

## Challenges and mitigation strategies for ferroelectric memories

**Fig. 10:**
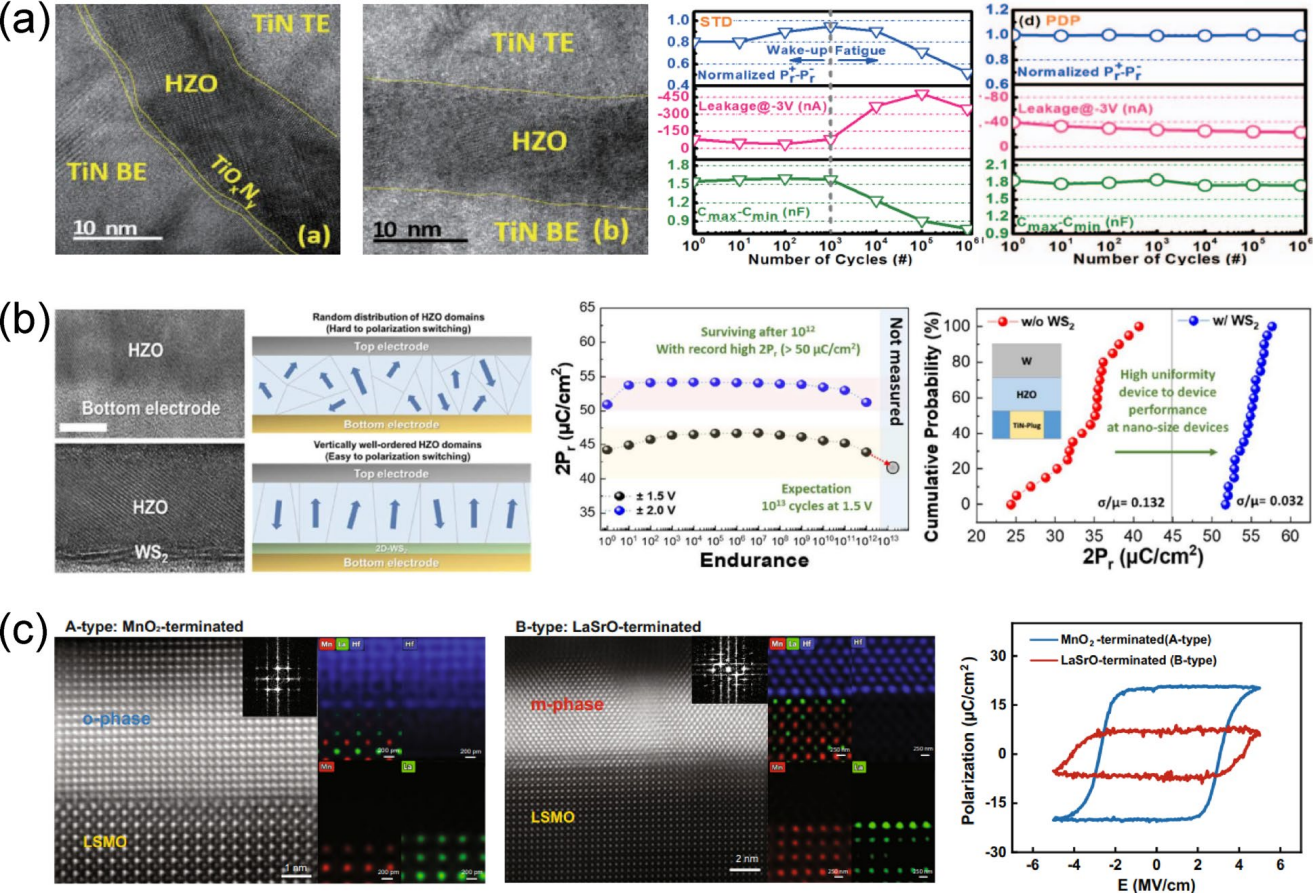
**a** Cross-sectional TEM image of a TiN/HZO/TiN capacitor after NH_3_ plasma treatment, showing the removal of the interfacial TiO_x_N_y_ layer between the bottom TiN electrode and HZO, resulting in a direct TiN/HZO interface (left panel), and endurance, leakage current, and capacitance characteristics before and after NH_3_ plasma treatment, demonstrating suppression of wake-up/fatigue effects, stable leakage, and negligible capacitance degradation over > 10^12^ cycles (right panel). Reproduced with permission from Ref [222]. Copyright 2017, IEEE. **b** Cross-sectional TEM images of HZO capacitors with and without a WS_2_ IL (left panel), endurance characteristics over > 10^12^ cycles with record-high polarization (2P_r_ > 50 μC/cm^2^) enabled by WS_2_-induced vertically ordered domains (middle panel), and cumulative probability distribution of 2P_r_ showing improved D2D uniformity at nanoscale with WS_2_ insertion (right panel). Reproduced with permission from Ref [228]. Copyright 2024, IEEE. **c** Atomic-resolution STEM images of 1.5 nm-thick epitaxial HZO films on LSMO with MnO-terminated (A-type) and LaSrO-terminated (B-type) interfaces (left panel), showing orthorhombic ferroelectric and monoclinic non-ferroelectric phases, respectively, with elemental mapping (insets) confirming distinct interfacial chemistry. *P*–*E* loops (right panel) exhibit strong ferroelectric switching for the A-type and suppressed polarization for the B-type, highlighting the role of interface termination in phase stabilization. Reproduced with permission from Ref [234]. Copyright 2023, Springer Nature

A key reliability concern in ferroelectric capacitors is the degradation of the electrode/ferroelectric interface [219]. In HZO films, this interface is susceptible to defect formation, oxygen vacancy accumulation, and undesirable chemical reactions during fabrication, leading to wake-up, fatigue, and leakage current increase [220]. As shown in the left panel of Fig. 10a, Chen et al. observed that the as-fabricated TiN/HZO interface contains a parasitic TiO_x_N_y_ layer formed during processing, which acts as a defect-rich interlayer that accelerates wake-up and fatigue [221]. They demonstrated that applying NH_3_ plasma treatment to the HZO surface prior to TE deposition effectively removes or suppresses this TiO_x_N_y_ layer, passivates oxygen vacancies, reduces defect density, and modifies interfacial bonding states. The right panel of Fig. 10a shows that this approach stabilized polarization from the initial cycles, suppressed the wake-up effect, and maintained stable switching characteristics with minimal fatigue even after 10^10^ cycles, while keeping leakage current consistently low. These results highlight that precise chemical engineering of the ferroelectric/electrode interface can substantially enhance endurance and operational reliability without the need for prolonged electrical conditioning [221–223].

Reliability in ferroelectric devices is strongly influenced by domain wall pinning and non-uniform polarization switching, which typically originate from randomly oriented ferroelectric domains in conventional polycrystalline HZO films [96, 97, 224–226]. The left panel of Fig. 10b presents cross-sectional TEM images revealing that in conventional structures without WS_2_, HZO exhibits randomly oriented domains, whereas the insertion of a monolayer WS_2_ between the BE and the HZO layer leads to vertically well-aligned ferroelectric domains [227]. The middle panel of the Fig. 10b contains schematic illustrations showing how this vertical ordering facilitates easier and more uniform polarization switching by suppressing domain wall pinning. The right panel of Fig. 10b displays endurance and cumulative probability plots, indicating that WS_2_-integrated HZO capacitors survive beyond 10^12^ switching cycles while maintaining a high 2*P*_r_ above 50 µC/cm^2^, along with a projected 10-year data retention at 85 °C. Moreover, the cumulative probability distribution shows a marked improvement in uniformity, with σ/μ reduced to 0.032 compared to 0.132 without WS_2_, underscoring the scalability and reliability of this interface engineering strategy for advanced nanoscale ferroelectric memory technologies.

A major challenge for hafnia-based ferroelectrics such as HZO is the degradation of ferroelectric properties when the film thickness is reduced to the nanometer scale. Most HZO thin films are polycrystalline, containing randomly oriented grains and grain boundaries. This microstructural inhomogeneity not only introduces structural defects and phase variability but also promotes the stabilization of non-ferroelectric monoclinic or tetragonal phases, effects that become increasingly pronounced below approximately 6 nm [66]. These issues, along with enhanced depolarization fields and increased interface energy, severely degrade polarization in the ultrathin regime. Accordingly, in addition to ALD, recent work has explored high-power impulse magnetron sputtering (HiPIMS) and pulsed-laser deposition (PLD). These routes offer different trade-offs in thermal budget, impurity incorporation, and control of ultrathin phases. HiPIMS provides a highly ionized metal flux that yields dense, low-impurity HfO_2_/HZO at comparatively low substrate temperatures and allows tuning of stoichiometry and oxygen vacancies via pulse width and reactive oxygen flow, although its usable process window is tighter and wafer-level uniformity and throughput are less mature than ALD [228–232]. By contrast, PLD affords strong texture control and, on suitable single-crystal substrates, epitaxial alignment of the HZO layer with the substrate lattice, which eliminates most grain boundaries and suppresses competing phases [233–238]. As shown in Fig. 10c, Shi et al. demonstrated that the atomic termination of the underlying perovskite electrode plays a decisive role in determining the stabilized phase in ultrathin epitaxial HZO [238]. The high-resolution STEM imaging and the corresponding selected area electron diffraction (SAED) pattern reveal that growth on MnO_2_-terminated LSMO results in an Orthorhombic phase HZO film. Elemental mapping confirms the termination configuration, and the atomically ordered lattice indicates the absence of grain boundaries, enabling robust ferroelectricity even at a thickness of 1.5 nm. The middle panel of the Fig. 10c shows that when the electrode surface is LaSrO-terminated, the HZO layer instead adopts a monoclinic phase. The right panel of Fig. 10c compares the *P*–*E* hysteresis loops of the two configurations, where the orthorhombic phase film on MnO_2_ termination exhibits a large remanent polarization, while the monoclinic phase film on LaSrO termination shows a markedly reduced polarization. These results highlight that precise control of interface termination during epitaxial growth can significantly aid in stabilizing the ferroelectric phase and enhancing the performance of ultrathin HZO capacitors.

While interface-oriented approaches such as plasma treatment, 2D interlayers, and termination-controlled epitaxy have demonstrated significant benefits, they can also raise concerns including lattice mismatch at inserted interfaces and wafer-scale variability. To mitigate these limitations, other material-intrinsic strategies have also been explored to improve the reliability of ferroelectric hafnia devices. One representative route is dopant engineering, where the incorporation of aliovalent elements such as Y, Si, or Zr induces lattice distortion and charge compensation, thereby stabilizing the orthorhombic ferroelectric phase in hafnia films [239]. Another effective approach is grain size engineering, achieved by adjusting ALD cycle ratios to control the crystallite dimensions of HZO films, which intrinsically promotes ferroelectric ordering even in highly scaled devices [53, 54]. Beyond these, oxygen vacancy management through ALD process parameter tuning has also been demonstrated: modifying oxidant pulse times during ALD directly tailors the vacancy concentration profile in HfO_2_ films, which in turn governs leakage and reliability characteristics [240]. Collectively, these intrinsic strategies provide scalable, CMOS-compatible pathways to enhance endurance, retention, and variability.

## Conclusion and prospects

This review has presented the historical development of ferroelectric materials, from the discovery of the first ferroelectric compounds to the emergence of Sc-doped AlN. Among these, doped HfO_2_ has emerged as the most prominent platform owing to its unique combination of scalability down to the few-nanometer regime, CMOS process compatibility, high coercive field, large bandgap, and the ability to be integrated using industry-established ALD processes with conventional electrode materials. Since the initial discovery of ferroelectricity in doped HfO_2_, extensive optimization efforts have been made to enhance its properties, including systematic engineering of dopant species and concentrations, control of HZO ALD cycle ratios, grain size engineering, annealing conditions, and electrode material and thickness tailoring. Through these advancements, the stability of the ferroelectric phase, polarization magnitude, and overall device performance have been markedly enhanced, positioning doped HfO_2_ as a leading candidate for CMOS-compatible and highly scalable ferroelectric integration.

Also, we reviewed four major device classes based on hafnia ferroelectrics: FeRAM, FTJ, FeFET, and FeCAP, detailing their operating principles, initial demonstrations, and subsequent key research milestones. Building on these milestones, subsequent research has focused on scaling to advanced technology nodes, enabling seamless integration within standard semiconductor process flows with improving endurance and retention. We also examined advanced integration strategies such as stacked vertical FTJ structures and FeNAND architectures for high-density 3D integration. The review further highlighted the growing application of hafnia-based ferroelectrics in in-memory computing, owing to their nonvolatility, fast switching speed, low operating voltage, and compatibility with advanced CMOS processes, which collectively enable energy-efficient and highly parallel computing architectures. We discussed their role in neuromorphic systems, where ferroelectric devices can emulate synaptic functionalities such as potentiation and depression, with the potential for linear and symmetric weight modulation and multi-state implementation. We also covered their application in hardware-based security, including TRNGs and PUFs, where intrinsic device variability such as domain polarization is harnessed as a robust entropy source. Additionally, we explored associative memories implemented with ferroelectric devices, achieving compact cell designs with low power consumption and high area efficiency.

Despite these advancements, hafnia-based ferroelectrics face critical reliability challenges. Wake-up, fatigue, retention loss, and interface instability remain key obstacles to long-term operational stability. Recent studies have shown that wake-up can be suppressed through optimized doping strategies in combination with capping or seed layer engineering, which minimize initial defect redistribution and stabilize the orthorhombic phase. Fatigue, arising from cumulative charge trapping and domain wall pinning under repeated cycling, can be alleviated by careful electrode selection and interface passivation layers that enhance long-term durability. Retention loss, primarily driven by depolarization fields and trap-assisted leakage, can be mitigated through dielectric scaling with ultrathin interlayers, insertion of 2D materials to improve screening, and strain engineering to reinforce temporal stability of polarization. Beyond these issue-specific solutions, interface engineering such as chemical treatments to remove defect-rich interlayers, incorporation of 2D materials for guided domain alignment, and termination-controlled epitaxial growth offers a unifying pathway to extend endurance, improve uniformity, and preserve polarization even in films thinner than 2 nm. Looking ahead, further advances in composition optimization, interface chemistry control, and microstructure engineering will be essential to fully exploit the intrinsic advantages of hafnia-based ferroelectrics. In parallel, innovations in circuit- and architecture-level design that leverage their non-volatility, fast switching, and multi-level capability could expand their application space beyond conventional memory into dense, low-power, and highly adaptive computing platforms. With the continued convergence of materials science, device engineering, and system architecture, hafnia-based ferroelectrics are positioned to enable a new class of energy-efficient nonvolatile memories and to drive the development of secure, high-speed, and intelligent in-memory computing systems.

## Data Availability

The datasets used and/or analyzed during the current study are available from the corresponding author on reasonable request.

## Abbreviations

1T–1C: One-transistor–one-capacitor
3D: Three-dimensional
A: Ampere
AFE: Antiferroelectric
Al_2_O_3_: Aluminum oxide
ALD: Atomic layer deposition
BE: Bottom electrode
BEOL: Back-end-of-line
BL: Bit line
C: Coulomb
C2C: Cycle-to-cycle
CAM: Content-addressable memory
CC: Cell contact
C_DEP_: Depletion capacitance
C_FE_: Ferroelectric capacitance
C_HCS_: Capacitance of high-capacitance state
C_LCS_: Capacitance of low-capacitance state
CMOS: Complementary metal–oxide–semiconductor
CMW: Capacitive memory window
CRP: Challenge–response pair
CTE: Coefficient of thermal expansion
C–V: Capacitance–voltage
D2D: Device-to-device
DRAM: Dynamic random-access memory
DSL: Defect shielding layer
E: Electric field
eV: Electron volt
EDX: Energy-dispersive x-ray spectroscopy
eNVM: Embedded non-volatile memory
ERS: Erase
F: Farad
FeCAP: Ferroelectric memcapacitor
FeFET: Ferroelectric field-effect transistor
FeNAND: Ferroelectric nand
FeRAM: Ferroelectric random-access memory
FDSOI: Fully-depleted silcon on insulator
FFT: Fast fourier transform
FN: Fowler–nordheim
FORC: First-order reversal curve
FTJ: Ferroelectric tunnel junction
HAO: Hafnium aluminum oxide
HD: Hamming distance
HCS: High-capacitance state
Hf: Hafnium
HfO_2_: Hafnium oxide
HKMG: High-k metal gate
HR-TEM: High-resolution transmission electron microscopy
HRS: High-resistance state
HZO: Hafnium zirconium oxide
Hz: Hertz
I: Current
IL: Interlayer
I_LRS_: Current of low-resistance state
I_HRS_: Current of high-resistance state
InZnO_x_: Indium zinc oxide
IoT: Internet of things
I–V: Current–voltage
J: Joule
K: Kelvin
LCS: Low-capacitance state
LRS: Low-resistance state
MANN: Memory-augmented neural network
MFIM: Metal–ferroelectric–insulator–metal
MFIS: Metal–ferroelectric–insulator–semiconductor
MFM: Metal–ferroelectric–metal
MFS: Metal–ferroelectric–semiconductor
ML: Match line
MOS: Metal–oxide–semiconductor
ms: Millisecond
nm: Nanometer
ns: Nanosecond
NVM: Non-volatile memory
P: Polarization
P–E: Polarization–electric field
PGM: Program
PL: Plate line
P_r_: Remanent polarization
PUF: Physical unclonable function
P–V: Polarization–voltage
R: Resistance
RTA: Rapid thermal anneal
s: Second
SA: Sense amplifier
SC: Sidewall contact
SEM: Scanning electron microscopy
SL: Source line / search line
STDP: Spike-timing-dependent plasticity
t: Time
TaN: Tantalum nitride
Ta_x_O_y_: Tantalum sub-oxide
T_c_: Curie temperature
TCAM: Ternary content-addressable memory
TE: Top electrode
TEM: Transmission electron microscopy
TER: Tunneling electroresistance
TiN: Titanium nitride
TRNG: True random number generator
V: Volt
V-FTJ: Vertical ferroelectric tunnel junction
VMM: Vector–matrix multiplication
V_th_: Threshold voltage
W: Tungsten
WL: Word line
XPS: X-ray photoelectron spectroscopy
Zr: Zirconium
ZrO_2_: Zirconium oxide
ΔV: Memory window / voltage shift
μm: Micrometer
Ω: Ohm
°C: Degree celsius
%: Percent
